# The *Bos taurus*–*Bos indicus* balance in fertility and milk related genes

**DOI:** 10.1371/journal.pone.0181930

**Published:** 2017-08-01

**Authors:** Parthan Kasarapu, Laercio R. Porto-Neto, Marina R. S. Fortes, Sigrid A. Lehnert, Mauricio A. Mudadu, Luiz Coutinho, Luciana Regitano, Andrew George, Antonio Reverter

**Affiliations:** 1 CSIRO Agriculture and Food, Queensland Bioscience Precinct, St. Lucia, Brisbane, Queensland, Australia; 2 School of Chemistry and Molecular Biosciences, The University of Queensland, Brisbane, Queensland, Australia; 3 Embrapa Agricultural Informatics, Campinas, Sao Paulo, Brazil; 4 Centro de Genomica Funcional ESALQ, University of São Paulo, Piracicaba, Sao Paulo, Brazil; 5 Embrapa Southeast Livestock, Rodovia Washington Luiz, São Carlos, Sao Paulo, Brazil; 6 CSIRO, DATA61, Ecosciences Precinct Brisbane, Brisbane, Queensland, Australia; Wageningen UR Livestock Research, NETHERLANDS

## Abstract

Numerical approaches to high-density single nucleotide polymorphism (SNP) data are often employed independently to address individual questions. We linked independent approaches in a bioinformatics pipeline for further insight. The pipeline driven by heterozygosity and Hardy-Weinberg equilibrium (HWE) analyses was applied to characterize *Bos tauru*s and *Bos indicus* ancestry. We infer a gene co-heterozygosity network that regulates bovine fertility, from data on 18,363 cattle with genotypes for 729,068 SNP. Hierarchical clustering separated populations according to *Bos tauru*s and *Bos indicus* ancestry. The weights of the first principal component were subjected to Normal mixture modelling allowing the estimation of a gene’s contribution to the *Bos taurus-Bos indicus* axis. We used deviation from HWE, contribution to *Bos indicus* content and association to fertility traits to select 1,284 genes. With this set, we developed a co-heterozygosity network where the group of genes annotated as fertility-related had significantly higher *Bos indicus* content compared to other functional classes of genes, while the group of genes associated with milk production had significantly higher *Bos taurus* content. The network analysis resulted in capturing novel gene associations of relevance to bovine domestication events. We report transcription factors that are likely to regulate genes associated with cattle domestication and tropical adaptation. Our pipeline can be generalized to any scenarios where population structure requires scrutiny at the molecular level, particularly in the presence of *a priori* set of genes known to impact a phenotype of evolutionary interest such as fertility.

## Introduction

Genotype data from high-density single nucleotide polymorphism (SNP) arrays serves as a starting point for many genomic analyses as they can reflect a wide range of processes [1–3]. SNP data have been used to characterize linkage disequilibrium and estimate effective population size [4,5], to perform genome-wide association studies [6–8], to compress genomes and highlight regions of evolutionary interest in humans and livestock species [9–11], to study the genetic variants of common diseases [12–14], and to identify population structure and signatures of selection [5,15–18]. These numerical approaches are employed independently to address specific questions. Formally linking them in a computational routine can drive discovery. Population assignment at the DNA level can inform genotype-phenotype associations, because phenotypes of each population (or lineage) are distinct. Herein, we propose a computational routine to maximize the use of SNP data in a comparative genomics framework.

A typical use of SNP data for population genetics involves computation of percentage of heterozygosity (HET), fixation index (F_ST_), and principal component analysis (PCA) [3]. The HET values serve as a summary of genotype data and provide first-hand information about the genetic diversity within a population. Related measures such as extended haplotype homozygosity (EHH) [19] and its variants have been used to identify selective sweeps and signatures within cattle breeds [20–23]. A literature gap is the exploration of HET values across genetically diverse cattle breeds. Computation of HET from SNP data could facilitate the discrimination of breeds with divergent ancestry (that is, sub-species of cattle: *Bos indicus* and *Bos taurus*). We proposed that gene ancestry can be calculated by computing the average HET of its SNP.

First attempts to classify livestock breeds using genetic markers were originally based on microsatellites [24–27] and most analysis included a few hundred animals and a handful of breeds. The Bovine HapMap Consortium [28] interrogated 37,470 SNP in 497 animals and used PCA to elucidate the genetic structure of diverse breeds. PCA was used to measure genetic divergence in *Bos indicus* and *Bos taurus* cattle [29,30] and to inform machine learning methods to predict cattle ancestry [29,31]. We distinguish our current work by developing new methods and expanding the dataset to include hundreds of animals per breed. We used PCA as a starting point to identify genes that have discriminatory power to identify cattle population as *Bos indicus* or *Bos taurus*. Then clustering methods were applied to average HET values to prove that our measure of gene ancestry is able to segregate cattle breeds according to known lineages, similarly to PCA. As HET values differ across breeds, we noticed a striking contrast between the set of genes that have high/low HET values in each breed. Gene ancestry was linked to biological processes in Gene Ontology enrichment analyses followed by annotation of gene attributes (whether a gene is a transcription factor, expressed in tissue-specific manner, codes a secreted protein, or codes kinases). Finally, we investigated if genes relevant to breed differences could interact with genes associated to fertility or lactation by building gene network based on average HET correlations.

## Results and discussion

### Overview of the bioinformatics pipeline

**Fig 1:**
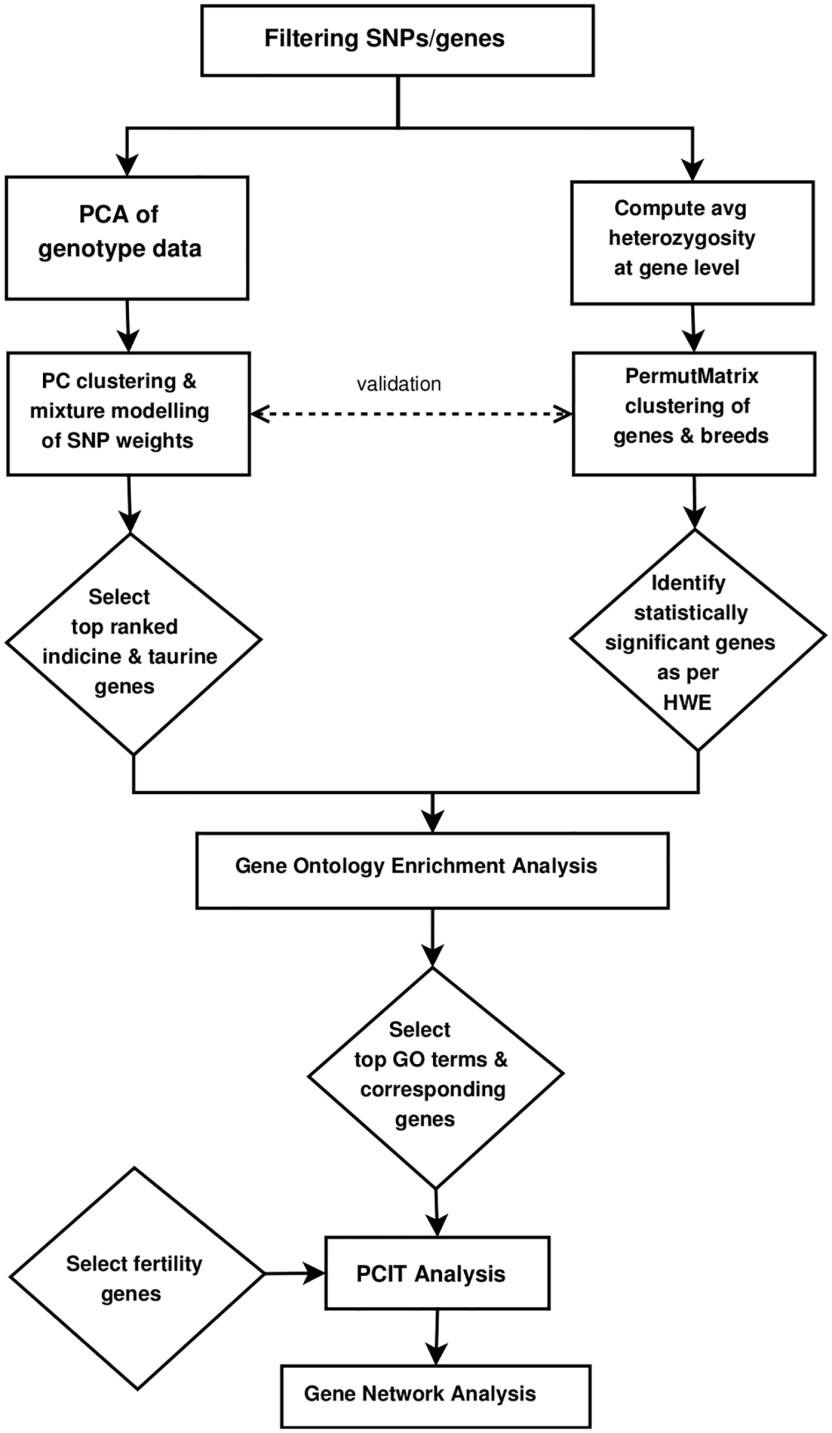
Flowchart of the pipeline for exploratory analysis of the effects of heterozygosity in the bovine genome.

Our approach to analysing the genotype data of the various cattle breeds is schematically illustrated in the flowchart of Fig 1 and summarized in six steps: 1. Data pre-processing to select animal populations and genotypes for SNP in autosomal chromosomes within 1 kb of a known protein coding gene; 2. Principal component analysis (PCA) of the genotype data to characterize population structure; 3. Computation of the gene-level heterozygosity followed by clustering analysis to dissect population structure and selection of statistically significant genes based on deviation from Hardy-Weinberg equilibrium (HWE) values; 4. Perform Gene Ontology (GO) enrichment analysis on *two gene lists*–one derived from *Bos indicus* vs. *Bos taurus* content and another from HWE deviation; 5. Generation of a gene co-heterozygosity network using partial correlation and information theory [32] for candidate genes from the two lists, alongside fertility-related genes and milk-related genes from previous studies; 6. Analysis of the network structure and determination of the key genes. These six steps are detailed in S1 Text.

### Principal component and heterozygosity analyses reveal population structure in accordance to *Bos indicus* and *Bos taurus* ancestry of cattle breeds

**Fig 2:**
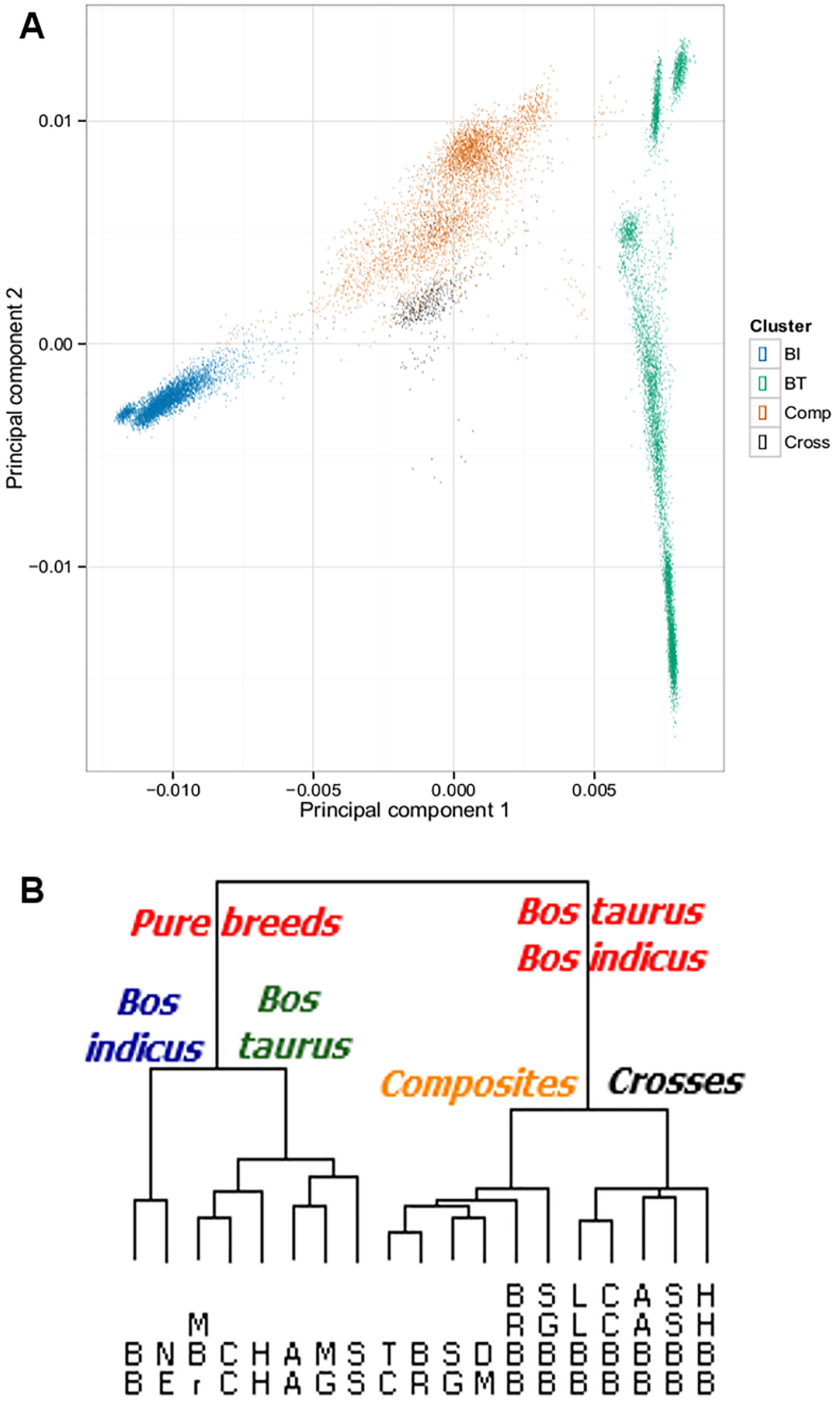
**(A)** Principal Component Analysis of SNP genotypes corresponding to cattle breeds grouped based on their lineage. Left illustrates *Bos indicus* (BI), Middle shows *Bos taurus—Bos indicus* (cross-breeds and composite breeds), and Right corresponds to *Bos taurus* (BT); **(B)** Hierarchical clustering analysis of heterozygosity of 8,631 genes across the 19 cattle breeds produces a dendrogram showing the clustering of breeds consistent with their respective lineages.

We performed a principal component analysis (PCA) of the genotype data (246,864 SNP) for 18,363 cattle of 19 breeds. A clear separation between the breeds based on their lineage is evident from PCA analyses (Fig 2A). The first two principal components explained 21.8% (PC1) and 2.3% (PC2) of the variation. We observed the pure *Bos indicus* breeds (BI) on the extreme left and the pure *Bos taurus* breeds (BT) on the extreme right of the PC1 spectrum. The middle region of the plot depicts the cattle corresponding to the *Bos taurus-Bos indicus* crossbreeds. These observations are consistent with documented knowledge of cattle history [28,33]. The crossbreeds LLBB, CCBB, AABB, SSBB, HHBB have similar genetics and clustered together (black cluster in Fig 2A). Similarly, the tropically-adapted breeds TC, BR, DM, and SG are clustered together (orange cluster in Fig 2A).

*Bos taurus* breeds are genetically more conserved compared to the pure *Bos indicus* breeds–the *Bos taurus* breeds showed higher LD (r2 = 0.45) than their indicine (r2 = 0.25) and composite (r2 = 0.32) counterparts. This higher LD in taurine breeds was attributed to a smaller effective population size and a stronger bottleneck during breed formation [5].

A relative smaller variation within the *Bos taurus* breeds was observed, largely scattered along PC2 (see Fig 2A). In contrast, the *Bos indicus* breeds have larger variation along PC1 and we observe a gradual transition into the *Bos taurus-Bos indicus* breeds, consistent with previous findings [33]. The link between PC1 and *Bos indicus* content has motivated us to formally ascertain this relationship by computing the contribution of individual SNP to the *Bos indicus* content of the cattle. Recall that each principal component is a weighted linear combination of the features (SNP) in the data set. As part of PCA, we obtained the SNP weights for each of the principal components and as such, the importance of the SNP to each principal component.

We considered PC1 and analysed the SNP weights along this vector with an expectation, based on Fig 2A, that the pure *Bos indicus* breeds would have a negative value, the pure *Bos taurus* breeds would have positive values, and the *Bos taurus-Bos indicus* breeds would have a combination of positive and negative values. The empirical distribution of the SNP weights followed two distinct modes that required a mixture model with two normal distributions to quantify the contribution of the SNP to the *Bos indicus* content in cattle (S2 Text). Membership of 31% of SNP to *Bos indicus* and 69% to *Bos taurus* components was estimated (S1 Fig). We provided our entire list of 8,631 genes and their contributions to the indicine and taurine components of the bovine genome in S3 Text.

Hierarchical cluster analysis with respect to HET values was carried and also revealed the separation of cattle into distinct groups based on their ancestry and breed type (Fig 2B). The first partition in the hierarchy corresponds to purebreds and crossbreds towards the left and right, respectively. Within each pure versus cross-bred partition, we observed a remarkable separation based on the lineage of breeds. BB and NE (pure *Bos indicus*) have their own cluster while the breeds MBr, CC, AA, HH, MG, and SS are clustered together (pure *Bos taurus*). Among the cross-breeds with *Bos taurus- Bos indicus* lineage, we observed that the cross-breeds LLBB, CCBB, AABB, SSBB, and HHBB are clustered together. Similarly, the composite breeds TC, BR, DM, SG, BRBB, and SGBB are clustered together. These results align with those reported above for the PCA method [33]. The clustering method was able to detect this hidden population structure based only on the heterozygosity values at the gene level.

**Fig 3:**
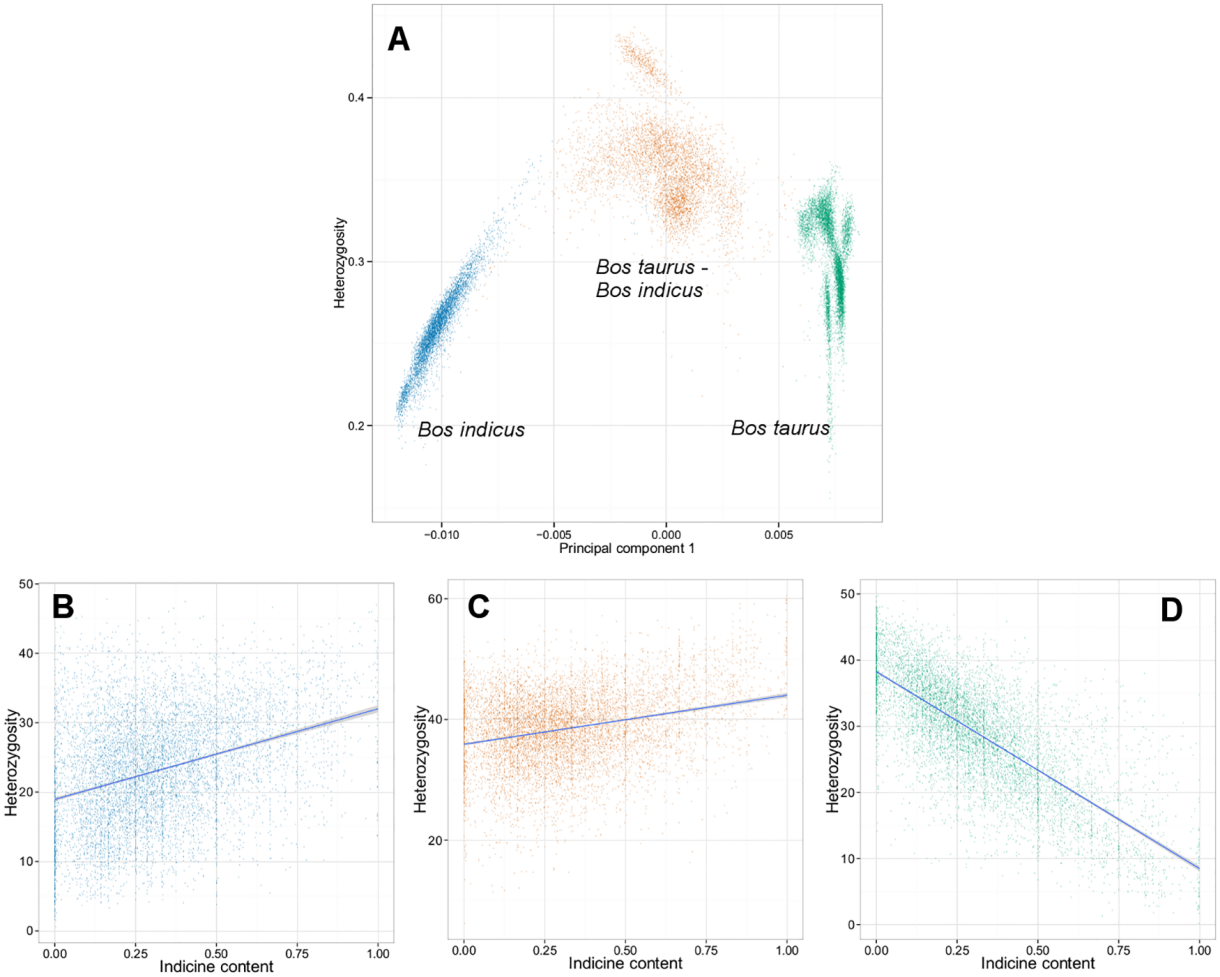
Relationship between heterozygosity and Bos indicus content at the animal and gene-level derived from PC1. **(A)** Heterozygosity against PC1 in each animal results in an inverted-V pattern; **(B)** Heterozygosity at gene-level based on lineage for *Bos indicus*; **(C)** *Bos taurus–Bos indicus*; and **(D)** *Bos taurus*.

Heterozygosity and *Bos indicus* content were correlated metrics at the animal level and at the gene level within lineages (Fig 3). At the animal level, we found a strong non-linear relationship between the PC1 values and heterozygosity. This inverted V pattern has been recently reported by Samuels et al. [34] with various human populations. Its recapitulation here (Fig 3A) with beef cattle suggests some universal law by which heterozygosity alone governs the principal population structure in a genetically diverse sample. For the 8,631 genes under consideration, we observed a strong linear relationship between a gene’s heterozygosity and its contribution to *Bos indicus* content within the *Bos taurus* lineage (Fig 3D; Pearson correlation, r = –0.74), while the correlation strengths with the *Bos indicus* (Fig 3B) and *Bos taurus-Bos indicus* (Fig 3C) lineages are 0.35 and 0.28, respectively. The negative sign indicates that genes with low heterozygosity contribute significantly to the *Bos indicus* content in *Bos taurus* breeds. On the other hand, the positive correlations observed for the *Bos indicus* and *Bos taurus- Bos indicus* lineages, indicate that an increase in the heterozygosity of genes relates to an increase in the net *Bos indicus* content.

Heterozygosity clustering detection was possible even when only 86 fertility-related (FE) genes were used in the analyses (S2 Fig). For FE genes that were common across at least three publications [35–37],we present heterozygosity results and *Bos indicus* content in Table 1.

**Table 1:** Fertility-related genes common across the literature sources and their heterozygosity and *Bos indicus/Bos taurus* contributions.

Gene	Number of SNP	Functional Attributes^A^	Heterozygosity (Lineage)			Posterior Probability (gene’s contribution)	
			*Bos indicus*	*Bos taurus*	*Bos taurus–Bos indicus*	*Bos indicus*	*Bos taurus*
ADH6	13	TS	23.81	40.56	45.42	27.02	72.98
E2F3	26	TF	13.81	30.44	30.66	8.62	91.38
ELF5	17	TF, TS	14.38	37.20	44.55	17.64	82.36
ETS1	24	TF	17.61	36.80	41.89	20.78	79.22
ETV6	50	TF	17.23	33.85	37.88	19.16	80.84
LHX4	12	TF	17.31	21.74	28.60	25.17	74.83
OVGP1	7	SE	32.28	40.23	43.98	0.55	99.45
PPARG	18	TF	19.70	34.74	42.04	16.97	83.03
PPP3CA	94	TS	25.43	29.59	36.96	26.34	73.66
SOX5	164	TF	20.23	25.81	37.03	30.79	69.21
TSHR	44	TS, SE	26.47	29.91	37.05	33.12	66.88

^A^TF = transcription factor; TS = tissue specific; SE = secreted.

### Analysis of indicine and taurine content in fertility and milk related genes

We collected 86 fertility genes as detailed in S1 Text. The milk related genes were sourced from the Cattle component (http://www.animalgenome.org/cgi-bin/QTLdb/BT/genesrch?gwords=milk) of the Animal QTL database [38] and from literature [39,40]. We collected 231 milk related genes and 125 of these were represented in our entire list of 8,631 genes.

**Fig 4:**
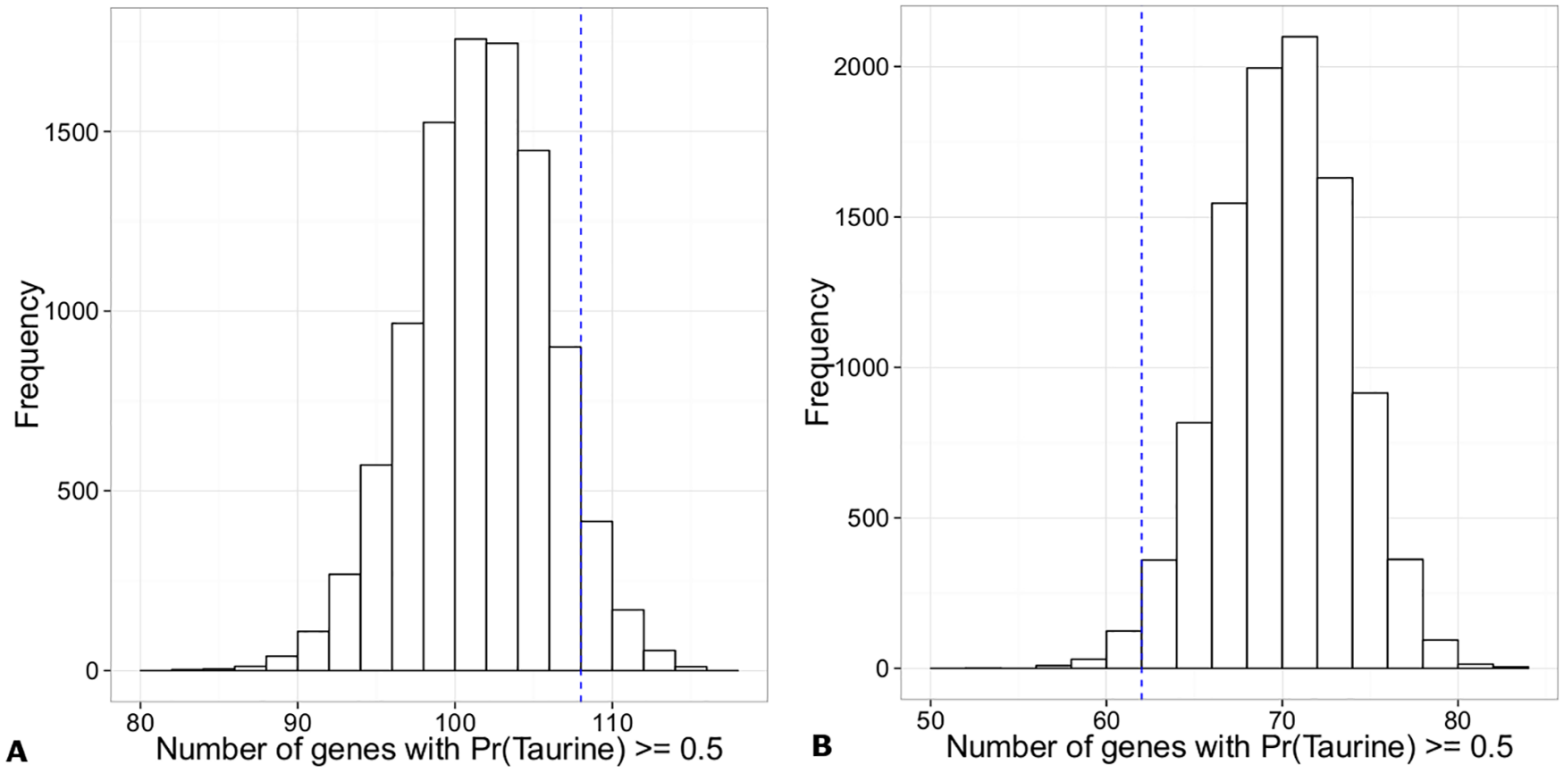
Distribution of the number of genes that have a Pr(Taurine) > = 0.5 after conducting 10,000 permutation tests. **(A)** Milk related genes; and **(B)** Fertility related genes. The vertical blue line indicates the observation of 108 milk related genes and 62 fertility related genes that showed a Pr(Taurine) > = 0.5 in our selected list of 8,631 genes.

For both the fertility and milk related genes, we computed their memberships to the indicine and taurine components. For the milk related genes, we observed that 108 (out of 125) genes have a posterior probability of at least 0.5 of having a taurine origin. To prove that this has not occurred by chance alone, we conducted a permutation test with 10,000 experiment trials. In each experiment, we randomly sampled 125 genes from the 8,631 genes and checked how many of them have at least 0.5 posterior probability of belonging to the taurine component. The corresponding histogram is shown in Fig 4A. From the distribution, we notice that 108 belongs to the 93.5^th^ percentile, which suggests that there is only about 6% chance that the 108 genes belong to the taurine component *by chance* alone. This suggests that the milk related genes are strongly associated with the taurine axis and this has been previously discussed in the literature [41–44]. Thus, we provided a proof-of-concept where our designed methodology of dissecting the bovine genome is able to identify genes that contribute to a phenotype of interest (milk related).

For the list of fertility genes, 62 out of 86 genes had a posterior probability of at least 0.5 of belonging to the taurine component. However, the permutation test indicated that 62 corresponds to the 16^th^ percentile as shown in Fig 4B. This suggests that the fertility genes are not associated with the taurine axis but are strongly associated with the indicine component of the bovine genome. This novel discovery could yield new insights into the evolution of fertility traits in the bovine genome.

### Analysis of *Bos indicus* content by chromosome

**Fig 5:**
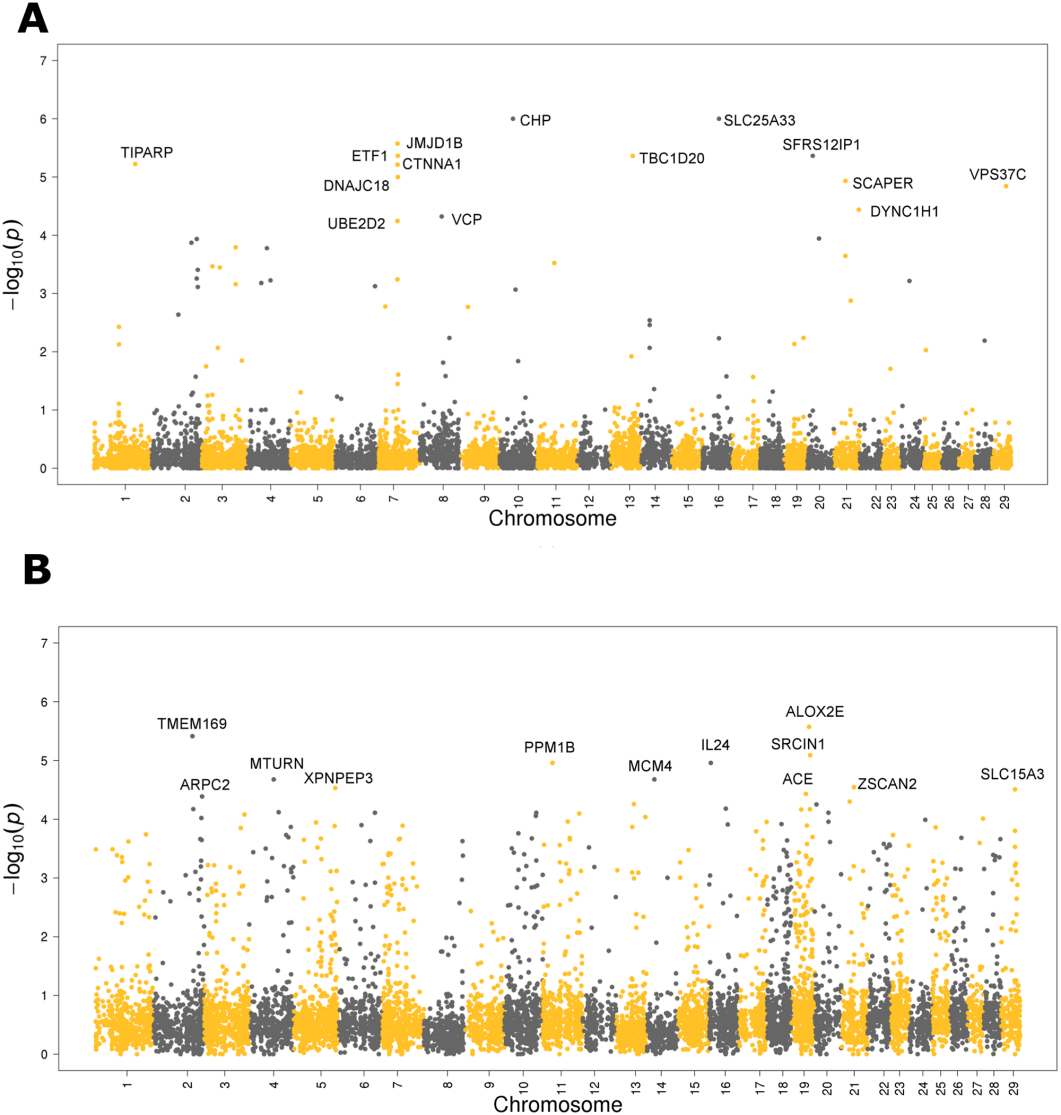
Genome-wide distribution plots depicting the highly contributing genes to the *Bos indicus* and *Bos taurus* contents in the bovine genome. Each point corresponds to a gene from our list of 8,631 genes along the genome. The likelihood of a gene being of *Bos indicus* or *Bos taurus* origin is plotted along the Y-axis. **(A)** Genes with high *Bos indicus* (low *Bos taurus*) content **(B)** Genes with low *Bos indicus* (high *Bos taurus*) content.

The contribution of each of the genes to the *Bos indicus* and *Bos taurus* components allowed us to compute a chromosome’s contribution by averaging the posterior probabilities across the genes within a chromosome. The resulting genome-wide distribution plots are shown in Fig 5, where each point corresponds to a gene from our list of 8,631 genes sorted on the x-axis by genome map position. The y-axis indicates the–*log(p)*, where *p* is the posterior probability, *m*_1_ for Bos indicus and *m*_2_ for Bos taurus in Equation 1 (S1 Text). We observed there are fewer genes that stand out with respect to their contribution to the *Bos indicus* content (Fig 5A), while there are a greater number of genes contributing to the *Bos taurus* content (Fig 5B). A more detailed analysis, revealed 14 genes with a significant contribution (*–log(p)* > 4) to the Bos indicus content (*TIPARP*, *JMJD1B*, *ETF1*, *CTNNA1*, *DNAJC18*, *UBE2D2*, *VCP*, *CHP*, *TBC1D20*, *SLC25A33*, *SFRS12IP1*, *LOC100140107*, *LOC537748*, *and VPS37C*). One enriched GO term from this list is GO:0071822 (Protein complex subunit organization) with a FDR p-value = 0.00505. Worth mentioning is *DNAJC18* (DnaJ heat shock protein family member C18) due to its recently reported association with heat stress in contrasting *Bos taurus* and *Bos indicus* cattle [45,46]. Also noteworthy is *SLC25A33* estimated to have a contribution to Bos indicus of 100% and encoded at 44.8 Mb of BTA16 in a hard-sweep region recently reported to be shared among four *Bos taurus* breeds [23] and possibly related to the initial cattle domestication events.

Similarly, we found 29 genes with statistically significant contribution to Bos taurus content (*–log(p)* > 4): *TMEM169*, *ARPC2*, *SRRM1*, *EPHA8*, *UTP11L*, *LOC615685*, *INHBA*, *XPNPEP3*, *HNRNPD*, *ACOT2*, *EIF2B2*, *PPM1B*, *CIZ1*, *CHGB*, *ADNP*, *MCM4*, *IL24*, *GPR157*, *ALOX12E*, *LOC535629*, *ACE*, *LOC506185*, *FASN*, *BNIP1*, *LOC782185*, *ZSCAN2*, *CLK3*, *NR1D2*, and *SLC15A3*. From this list, we highlight *FASN* (fatty acid synthase) and *INHBA* (Inhibin, beta activin beta-A chain). Ample evidence from the Animal QTL database [38] (http://www.animalgenome.org/cgi-bin/QTLdb/index) suggests the presence of QTL in the coding region of *FASN* associated with body weight, marbling and milk fat yield in cattle. The same source documents *INHBA* as harbouring QTL for semen volume, sperm counts and motility. Fortes et al. [47] propose SNP associated with serum levels of Inhibin in Brahman bulls as an early biomarker of sexual development. This same QTL was absent when Tropical Composite bulls were subject to GWAS for the same phenotypes [48]. These contrasting GWAS results reinforce the idea that *INHBA* polymorphism segregation and association with reproduction differs according to *Bos indicus* content of each breed.

### Analysis of heterozygosity at the chromosome level

**Fig 6:**
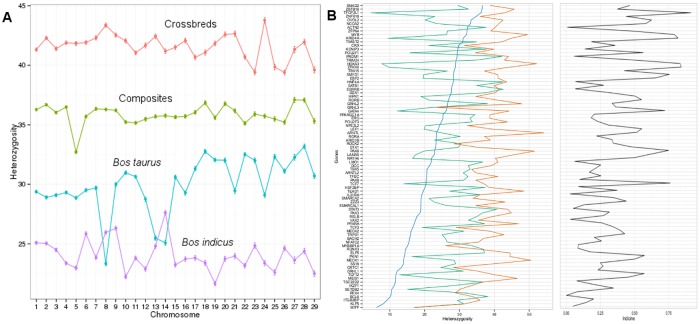
**(A)** Average chromosome heterozygosity across the four cattle lineages. **(B)** Variation of heterozygosity across the cattle lineages (blue, orange and green for *Bos indicus*, *Bos taurus–Bos indicus* and *Bos taurus*, respectively) for the list of 84 network genes (out of 1,284) that are TF and fertility related as well as at least one of the other functional attributes (TS, SE, KI). The right panel shows the contribution to the indicine content for the same set of genes.

In addition to the analysis of heterozygosity at the gene level, the average heterozygosity across the 29 autosomal chromosomes in the bovine genome reveals a striking contrast in the heterozygosity across the four different lineages (Fig 6A). The *Bos indicus* lineage has the least heterozygosity while the cross-breeds have the highest heterozygosity across the genome. Within each lineage, there appeared to be some chromosomes with dramatic changes in heterozygosity relative to the other chromosomes. For instance, BTA14 in *Bos indicus* has a greater heterozygosity and has a relatively large value when compared to its immediate neighbours which indicates that the BTA14 may be an important locus for introgression of *Bos taurus* genes. In fact, the importance of BTA14 and its role in milk production and ovulation rate has been well documented in the literature [49–51]. Furthermore, there is the age at first calving QTL on BTA14 that was detected in Nelore cattle [52–54].

Similarly, BTA8, BTA13, and BTA14 in *Bos taurus* contain the lowest average heterozygosity. Further, BTA5 within the composite breeds contains the lowest heterozygosity, while BTA27 and BTA28 contain the highest heterozygosity. BTA13, BTA14 and BTA28 have been reported to harbour QTL for carcass traits [55] while QTL on BTA5 are known to have a pronounced effect on reproductive efficiency in cattle [56–58]. The heterozygosity analysis brings to light some of the important regions in the cattle genome for breed discrimination.

### Gene Ontology (GO) enrichment analysis

The exploration of possible biological functions inherent in candidate gene lists is often done by a GO enrichment analysis [59]. The objective is to identify the set of genes which are significantly overrepresented in a target set of genes relative to a background set of genes. For each cattle breed, we have a target list of genes which deviate significantly from HWE. These gene lists are important as they could potentially be the variants which give cattle sub-species their distinctive *Bos indicus* or *Bos taurus* phenotypes. As a result, we have 19 target lists corresponding to each cattle breed. We conducted 19 separate analyses and collected the enriched GO terms statistically overrepresented up to a p-value level of 0.1%. This resulted in 142 GO terms, each occurring a maximum of three times with 7 of them (about 5%) occurring exactly thrice. These 7 GO terms together correspond to 1,193 genes in our list of 8,631 genes (Table 2). According to this analysis, genes involved with the regulation of developmental processes are overrepresented in genome regions that deviate from HWE. It is possible to extrapolate that the formation of cattle subspecies by phenotypic selection, has afforded particular importance to genome regions involved with fine-tuning the development of tissues and organs during development.

**Table 2:** Overrepresented GO terms in cattle genome regions which deviate from HWE corresponding to a total of 1,193 genes. Each GO term is enriched thrice.

GO Term	Description	p-value	Genes
GO:0007155	cell adhesion	1.40e-04	388
GO:0022610	biological adhesion	1.51e-04	389
GO:0031344	regulation of cell projection organization	1.94e-05	258
GO:0043547	positive regulation of GTPase activity	8.94e-05	222
GO:0050793	regulation of developmental process	3.62e-04	746
GO:0051960	regulation of nervous system development	3.27e-04	302
GO:2000026	regulation of multicellular organismal development	2.93e-04	576

We also considered the list of top ranked genes based on their contribution to the *Bos indicus* and *Bos taurus* content We selected those genes which have a membership of at least 95% to the *Bos indicus* and *Bos taurus* clusters (see Methods section and S1 Text for details). This resulted in 64 and 718 genes with high contribution to the *Bos indicus* and *Bos taurus* clusters, respectively that were targeted in two separate GO enrichment analyses. A striking enrichment of gene annotation terms associated with RNA splicing and mRNA processing was observed (Table 3). It is tempting to consider the possibility that the post-transcriptional processing machinery is overrepresented among the genes which potentially discriminate between the *Bos taurus* and *Bos indicus* subspecies. Post-transcriptional processing is an important element of gene regulation and could well contribute to sub-species differences.

**Table 3:** Overrepresented GO terms among a list of cattle genes which significantly contribute to the Bos indicus/Bos taurus content of cattle genomes, corresponding to a total of 52 genes. Each GO term occurs once.

GO Term	Description	p-value	Genes
GO:0000398	mRNA splicing, via spliceosome	4.85e-05	18
GO:0002082	regulation of oxidative phosphorylation	5.95e-04	2
GO:0002467	germinal center formation	2.06e-04	4
GO:0002544	chronic inflammatory response	6.53e-05	5
GO:0006397	mRNA processing	8.55e-04	25
GO:0006890	retrograde vesicle-mediated transport, Golgi to ER	7.05e-04	10
GO:0008380	RNA splicing	4.43e-05	24
GO:0071826	ribonucleoprotein complex subunit organization	4.91e-04	15

### Selection of genes for network analysis

Given that fertility phenotypes are an important consideration in the formation of domestic breeds, we constructed a co-heterozygosity network in order to further scrutinise the potential role of fertility-related genes in regions of high heterozygosity. The genes included in the co-heterozygosity network were selected from three possibly overlapping lists: 1) Genes based on their deviation from HWE; 2) Genes based on their significant contribution to the *Bos indicus/Bos taurus* content; and 3) Fertility (FE) related genes.

We obtained 1,193 genes not in HWE and 52 genes which predominantly contributed to the Bos indicus and Bos taurus content. These two lists were combined with the 86 FE genes sourced from the literature. The three lists contained 1,284 unique genes that were further categorized based on their functional attributes: transcription factors (TF), tissue-specific (TS), secreted (SE) and kinases (KI). We identified 84 out of the 1,284 genes that are TF and were also classified as either TS, SE, KI or FE. The variation of heterozygosity in these set of 84 TF and across the *Bos taurus* (BT), *Bos indicus* (BI) and *Bos taurus—Bos indicus* (BTI) lineages is shown in Fig 6B. The number of network genes that overlap with TF include 73 genes that are expressed in a tissue-specific manner, 10 genes that code for proteins that could potentially be secreted outside the cytoplasm (*ARNTL*, *GRHL3*, *IL31RA*, *KCNIP3*, *LAMA5*, *MEIS1*, *SATB1*, *SMARCA2*, *TCF12*, *TRIM24*), 3 genes that code for kinases (*HIPK1*, *PKN1*, *ROCK2*), and 61 genes that are fertility related.

### Gene co-heterozygosity network

We generated a gene co-heterozygosity network using the PCIT algorithm to identify significant connections based on correlated heterozygosity values for the 1,284 genes. These correlations were used to establish gene to gene edges in the network inference. This approach is able to point to genes for which the *Bos taurus* or *Bos indicus* origin is particularly crucial for animal performance. Imposing a correlation threshold of 0.95, we obtained a sub-network of 328 genes with 1,098 significant connections. Other thresholds were explored and further details are provided in S2 Text. We observed that the degree distribution on a logarithm scale follows a scale-free network, shown in S6 Fig (correlation of 0.85 and p-value of 2.2 x 10^−16^). The maximum degree is 47 and corresponds to the *SPEN* gene, which is a known transcriptional regulator [60–62]. The contribution to *Bos indicus* for *SPEN* was estimated at 64.80% placing it in the top 9% of all 8,631 genes.

We observed a significantly higher *Bos indicus* content for the 86 FE genes as compared to the remaining 1,198 network genes. This was attributed to fertility genes being under strong selection among the various cattle lineages [63]. Further exploitation of FE and their roles in the predicted co-heterozygosity network are offered in the S2 Text.

Some of the interesting genes that are present in this network include *BRCA1* which is involved in bovine mastitis [64,65], *MCF2L* which is known to play a critical part in joint tissue development in humans [66], *FOXP2* which is a TF required for proper development of speech and language regions of the brain during embryogenesis in humans [67], *CREBBP* which acts as a binding protein that is important in embryonic development, growth control, and has been implicated in the embryo-placenta signalling in bovine embryos [68,69].

## Conclusion

Our pipeline can be generalized to any scenarios where population structure requires scrutiny at the molecular level, particularly in the presence of *a priori* set of genes known to impact a phenotype of evolutionary interest such as fertility.

## Methods

Animal Care and Use Committee approval was not required for this study because the data were obtained from existing phenotypic and genotype databases from the Cooperative Research Centre for Beef Genetic Technologies (“Beef CRC”; http://www.beefcrc.com).

### Data collection and pre-processing

Genotypes from 17,867 cattle representing 18 breeds were extracted from data previously reported [70]. Genotypes from 496 Nelore (NE), a pure *Bos indicus* breed were also included [71]. In total, 18,363 cattle of 19 breeds were studied (S3 Fig), of which Brahman and Nelore are *Bos indicus* (BI), six breeds are *Bos taurus* (BT), and eleven breeds are *Bos taurus-Bos indicus* composites (BTI). Cattle were genotyped with high-density chip (over 700,000 SNP). SNP mapped to sex chromosomes were removed from analyses as these behave differently with respect to HWE and genotypes were from both female and male cattle. We targeted a 1kb region surrounding known genes in order to capture SNP associated with protein-coding regions. Only genes that have at least the median number of corresponding SNP (six) were included in subsequent analyses. The final set comprised 246,864 SNP located in 8,631 genes.

### Principal component analysis, mixture modelling and gene ancestry

Principal component analysis (PCA) was performed with PLINK [72]. We extracted the weights of the first principal component (PC1) as it explains the maximum variability. Like others, we found that PC1 captured the *Bos indicus* component of cattle breeds [29,31,73]. It is conceivable that some SNP, mapped to certain genes, contribute more than others to the *Bos indicus* components. Bolormaa et al. [74] assigned chromosome segments to be of *Bos indicus* or *Bos taurus* ancestry using a weighted regression model of SNP allele frequencies. However, their method required pre-defined segment length and was not informed by PCA analyses. We used PCA output as a first step to project the data on to the maximum variable direction and used statistical machine learning and two-component mixture modelling to quantify the *Bos indicus* and *Bos taurus* content of a gene. Our method identifies gene ancestry and lists genes that contribute significantly to *Bos indicus* or *Bos taurus* ancestry. These genes harbour informative SNP for determination of cattle lineage. The two-component mixture used is detailed in S1 Text. Mixture parameters were estimated via maximum likelihood using EMMIX software [75]. After estimating mixture parameters, the contribution of each SNP to each component is given by its posterior probability of belonging to *Bos indicus* or *Bos taurus* components of the mixture model. SNP in coding region were collapsed to estimate gene contribution to *Bos indicus* ancestry, which implies that a gene has higher/lower probability of membership to *Bos indicus* or *Bos taurus* components.

### Heterozygosity, Hardy-Weinberg equilibrium and clustering of breeds

Percentage heterozygosity (HET) was computed for each SNP as the proportion of animals with a heterozygous genotype. HET was computed for each SNP and averaged over all the animals in a given breed. HET values of SNP were averaged to obtain gene level HET, in a cumulative text statistic [76] (S1 Text). Gene HET was used to cluster cattle breeds using PermutMatrix [77] software. Allelic frequencies were used determine deviation from HWE. A nominal *P*-value of 1% served as threshold to select genes with significant deviation from HWE.

### Gene Ontology (GO) enrichment analyses

We performed GO enrichment analysis using GOrilla [78,79] to aid biological interpretation of genes deems significant for each breed, based on SNP deviation from HWE. Genes with significant deviation were contrasted to background (all 8,631 genes studied). Ranked gene lists based on PC1 estimated contribution to *Bos indicus* and *Bos taurus* ancestry were also analysed with GOrilla.

### Functional attributes and bovine fertility related genes

Genes were catalogued as transcription factors (TF), genes which are expressed in a tissue-specific (TS) manner, genes encoding secreted proteins (SE), and kinases (KI) as shown in S1A Fig. TF were defined according to the Animal Transcription Factor Database (http://www.bioguo.org/AnimalTFDB/) [80]. TS were identified from the Tissue-specific Gene Expression and Regulation [81] in humans. SE were identified with the Human Protein Atlas [82], and KI with the Human Kinome database [83]. Human databases were used in the absence of similar cattle resources.

Genes associated with heifer puberty and other cattle fertility traits were retrieved from previous studies [35–37,84], shown in S4B Fig. The fertility-related genes were catalogued as per above criteria (TF, TS, SE, KI) and checked for overlapping with our list of 8,631 genes. In total, 1,157 genes related to fertility were in our dataset, shown in S4C Fig.

### Gene co-heterozygosity network

We inferred a co-heterozygosity gene network using the partial correlation and information theory (PCIT) algorithm [32] to identify significant edges. Genes that deviated significantly from HWE, genes that contributed strongly to *Bos indicus* or *Bos taurus* ancestry, and fertility-related genes were included in the network prediction. Cytoscape [85] was used to visualise and analyse the resulting network. A search algorithm was employed to locate the minimal trio of fertility-related genes that span the majority of the network topology [86].

## Supporting information

S1 Text Methods supporting information. (DOCX)

S2 Text Results supporting information. (DOCX)

S3 Text Contribution of 8,631 genes to the taurine or indicine component of the bovine genome. (TXT)

S1 Fig Mixture modelling of SNP weights along the first PC. Left and Right modes describe the *Bos indicus* and *Bos taurus* components, respectively. Red indicates the actual distribution of SNP weights, grey curves are the individual Normal distributions, and black curve is the mixture model obtained by combining the two Normal distributions.
(TIFF)

S2 Fig Hierarchical clustering of heterozygosity of 86 fertility related cattle genes (clustered as rows) that are used in our network analysis and the various cattle breeds (clustered as columns). The gradient from green to black to red correspond to low, medium and high heterozygosity.
(TIFF)

S3 Fig Frequency distribution of the cattle population (Y-axis) across the 19 cattle breeds (along X-axis). (TIFF)

S4 Fig Venn diagrams of subsets of the entire list of 8,631 genes and their functional attributes. **(A)** The list of 2,891 genes from the entire list of 8,631 genes that belong to the four main functional categories of transcription factor (TF), secreted hormones (SE), kinases (KI) and genes expressed in tissue-specific (TS) manner; **(B)** The subset of 1,157 fertility genes collected from the literature where Canovas, MThomas, Fortes_Rev and Fortes correspond to [35], [37], [84] and [36]respectively; **(C)** The same list of 1,157 fertility genes across the four functional attributes (excluding 10 genes that are not TF, TS, SE, KI).
(TIFF)

S5 Fig Distribution of correlation coefficients among the 1,284 network genes with red profile corresponding to significant correlations as determined the PCIT algorithm and stablishing edges in the network inference. (TIFF)

S6 Fig Distributions of the scale-free network post-PCIT analysis. **(A)** At a correlation cut-off of 0.90 comprising of 858 genes and 12,958 significant connections **(B)** At a correlation cut-off of 0.95 comprising of 328 genes and 1,098 connections.
(TIFF)

S7 Fig Variation of the indicine percentage across the different categories of transcription factor (TF), tissue specific (TS), secreted (SE), kinases (KI) and fertility (FE). All corresponds to the 8,631 genes in our analysis and PCIT corresponds to the 1,284 network genes. The only category for which significant differences exists (p-value < 0.01) in the indicine percentage is for fertility-related genes.
(TIFF)

S8 Fig Visualization of the gene co-heterozygosity network. The size of the node corresponds to the indicine content. The nodes in green are transcription factors and remaining nodes in the network are purple-coloured. Nodes that are triangle-shaped are fertility-related genes and others are denoted by circles: **(A)** PCIT network after applying a threshold of 0.90; **(B)** The network spanned by the trio of fertility related genes GATA4, NR1H4, VAX2; **(C)** The network spanned by the trio of fertility related genes ELF5, ROCK2, POU2F1.
(TIFF)
