# Supplementary material for: The Bos taurus–Bos indicus balance in fertility and milk related genes
Source: PLoS One. 2017 Aug 1;12(8):e0181930. doi: 10.1371/journal.pone.0181930 (PMC5538644; doi:10.1371/journal.pone.0181930)
Supplement: S1 Text — (DOCX) [file pone.0181930.s001.docx]

# S1 Methods Supporting Information

# The *Bos taurus*–*Bos indicus* balance in fertility genes

Parthan Kasarapu, Laercio R. Porto-Neto, Marina R. S. Fortes, Sigrid A. Lehnert, Mauricio A. Mudadu, Luiz Coutinho, Luciana Regitano, Andrew George and Antonio Reverter

# Methods

Genotype data from high-density single nucleotide polymorphism (SNP) arrays serves as a starting point for many genomic analyses as they can reflect a wide range of processes. We propose a computational routine to maximize the use of SNP data in a comparative genomics bioinformatics pipeline. This pipeline and associated methodology details are presented here as supporting information to the manuscript.

**Overview of the bioinformatics pipeline**

Our approach to analysing the genotype data of the various cattle breeds is schematically illustrated in the flowchart of Fig. 1 (main text) and summarized in six steps that are detailed subsequently. The six steps are:

1. Data pre-processing to select cattle populations and SNP genotypes for gene coding regions, that is within 1 kb of a known protein coding gene, in autosomal chromosomes.
2. Principal component analysis (PCA) of SNP genotypes to identify and characterize the population structure of cattle breeds. The SNP weights from the first principal component were used to quantify the contribution of each gene to *Bos indicus* genetics. Genes contributing the most to *Bos indicus* and *Bos taurus* nature of cattle were identified.
3. Compute the gene-level heterozygosity followed by clustering analysis to dissect the population structure of cattle breeds. Clusters obtained from hierarchical clustering analyses of HET values were compared to PCA results. Statistically significant genes in every breed based on their deviation from expected Hardy-Weinberg equilibrium (HWE) values were selected for Gene Ontology (GO) enrichment analyses.
4. Perform GO enrichment analysis on *two gene lists* – one derived from the contribution to the *Bos indicus*/*Bos taurus* content and another from HWE deviation – to identify the genes in overrepresented ontologies. This step aimed at understanding the biological function of genes with either *Bos indicus*, *Bos taurus* or breed specific ancestry.
5. Generation of a gene co-heterozygosity network using the partial correlation and information theory (PCIT) algorithm [1] to the above set of candidate genes from the two lists alongside fertility-related genes sourced from the literature [2-5].
6. Analyse the network structure and determine genes with high number of connections (network hubs) and investigate if these were transcription factors, expressed in a tissue-specific manner, coding for secreted proteins or coding for kinases. This step aimed at understanding the biological link between genes selected for ancestry significance and fertility-related genes in cattle.

Further methodology detail for each step is provided below.

**Data collection and pre-processing**

We considered the genotype data from the Beef CRC legacy database (<http://www.beefcrc.com/>) which was described by Bolormaa et al [6]. In brief, the raw data contains 18,450 cattle and we considered the top breeds based on their population frequency. This resulted in a total of 17,867 cattle belonging to 18 breeds (see S3 Fig). These are Brahman (BB; N = 5,040 cattle), Angus (AA; N = 2,459 cattle), Charolais (CC; N = 397 cattle), Hereford (HH; N = 751 cattle), Mixed-Breed (MBr; N = 2,854 cattle), Murray Grey (MG; N = 252 cattle), Shorthorn (SS; N = 876 cattle), Tropical Composite (TC; N = 1,788 cattle), Santa Gertrudis (SG; N = 1,566 cattle), Belmont Red (BR; N = 764 cattle), Droughtmaster (DM; N = 464 cattle), Brahman crosses with Limousin (LLBB; N = 245 cattle), Charolais (CCBB; N = 197 cattle), Belmont Red (BRBB; N = 84 cattle), Angus (AABB; N = 40 cattle), Hereford (HHBB; N = 30 cattle), Santa Gertrudis (SGBB; N = 30 cattle), and Shorthorn (SSBB; N = 30 cattle). Of these, BB is regarded as pure *Bos indicus,* while AA, CC, HH, MBr, MG, and SS are considered pure *Bos taurus*. The remaining TC, SG, BR, DM, LLBB, CCBB, BRBB, AABB, HHBB, SGBB, SSBB are *Bos taurus – Bos indicus* composites. Additionally, we included data from 496 Nelore (NE) *Bos indicus* breed from a previously described population [7]. Our subsequent analyses are performed on this pre-processed data set that now contains 18,363 cattle (17,867 + 496) corresponding to 19 breeds, of which two breeds are pure *Bos indicus* (BI), six are pure *Bos taurus* (BT), and 11 are *Bos taurus – Bos indicus* (BTI).

Initially, there were 729,068 SNP of which we considered those SNP located in autosomal chromosomes and mapped within 1kb up and downstream of a known gene. We ignored the SNP mapping to the sex chromosomes as these behave differently with respect to HWE and can produce aberrations due to the presence of both males and females in the population. We targeted a 1kb region surrounding known genes in order to capture SNP associated with protein-coding regions. This reduced the number of SNP to 267,305 corresponding to 17,012 genes. The median number of SNP which are mapped to a gene is six and ranges from 1 to 649. We further pre-processed the data so that we retained those genes that have at least the median number of corresponding SNP to ensure that the genes are minimally represented. This resulted in a final set of 246,864 SNP located in 8,631 genes.

**Principal Component Analysis, Mixture Modelling and Gene Contribution to *Bos indicus***

We used the functionality in the PLINK software (https://www.cog-genomics.org/plink2) [8] to perform the principal component analysis (PCA). We considered the first principal component (PC1) as it explains the maximum variability in the data and thereafter, extracted the weights of the 246,864 SNP to that component. Like others before us, we found that PC1 captured the *Bos indicus* component of cattle breeds [9-11]. It is conceivable that some SNP contribute more than others and this is related to the genetic variance which is explained by PC1 and those SNP/genes. In this regard, Bolormaa et al. [12] assigned chromosome segments to be of *Bos indicus* or *Bos taurus* origin using a weighted regression model of allele frequencies in SNP locations of the *Bos indicus* and *Bos taurus* cattle genome. However, this requires a pre-defined segment length and is not informed by prior analyses based on the principal components. We used the output from PCA as a first step to project the data on to the maximum variable direction and used statistical machine learning based mixture modelling to quantify the *Bos indicus* and *Bos taurus* content of a gene. In other words, we proposed a method to identify the set of genes that contributes significantly to either *Bos indicus* or *Bos taurus* and aids in the determination of the lineage of cattle.

Mixture modelling is a statistical method to construct a probability distribution by combining the effects due to several component probability distributions. We considered the Normal component distributions to model the probability of the SNP weights in PC1, which were best modelled using two component distributions. Formally, a two-component mixture is defined as

$$\Pr\left( x \right)=\underset{p_{1}}{\underbrace{w \mathcal{N}\left( x;\mu_{1},\sigma_{1} \right)}}+\underset{p_{2}}{\underbrace{\left( 1-w \right)\mathcal{N(}x;\mu_{2},\sigma_{2})}}$$

where $x$ corresponds to the data (SNP weights in PC1), $\Pr\left( x \right)$ is the probability distribution of the mixture, $w$ is the weight of the first component in the mixture, $\mu_{1},\mu_{2}$ and $\sigma_{1},\sigma_{2}$ denote the means and the standard deviations of the two Normal ($\mathcal{N}$) components, respectively. As part of statistical inference, the mixture parameters, that is, $w, \mu_{1},\mu_{2},\sigma_{1},\sigma_{2}$ are estimated via maximum likelihood using the EMMIX software [13].

After estimating the mixture parameters which best describe the 246K SNP weights, the contribution of each SNP to each components is given by its *posterior probability*, that is,

$m_{1} = \frac{p_{1}}{p_{1}+p_{2}}$ and $m_{2} = \frac{p_{2}}{p_{1}+p_{2}}$ (1)

# where *m*_1_ and *m*_2_ are the posterior probabilities of the given SNP to belong to the first and second component of the mixture, respectively. The values *p*_1_ and *p*_2_ constitute the two parts of Pr(*x*). Within each gene, the posterior probabilities (*m*_1_ values) of the SNP in its coding region were collapsed to estimate the genes contribution to the *Bos indicus* content in the bovine genome. Note that $m_{1}+m_{2}=1$, which implies that for a given gene, *m*_1_ and *m*_2_ correspond to the contributions (memberships) of that gene to the *Bos indicus* and *Bos taurus* components of the mixture model, respectively.

**Heterozygosity, Hardy-Weinberg equilibrium and Clustering of breeds**

We computed the percentage heterozygosity (HET) of the 246,864 SNP across all the cattle breeds. For each of the bi-allelic SNP, the percentage HET is computed as the proportion of animals with a heterozygous genotype. The resulting allelic frequencies were then used to compute the test statistic to determine its deviation from HWE. The test statistic follows a Chi-square distribution with 1 degree of freedom, that is, $X\sim\chi_{1}^{2}$

The percentage HET is computed for each SNP and averaged over all the animals in a given breed population. For a given gene, we computed an average of the average HET values to obtain the gene level HET. Since we also compute the $\chi^{2}$ statistic at a SNP level, it is converted to a gene level statistic as follows. Consider a gene $g$ with $j$ associated SNP and $j$ test statistics, $X_{1},...,X_{j}$. The cumulative test statistic $X(g)$is given by

$$X(g)=X_{1}+...+X_{j}$$

Where $X(g)\sim\chi^{2}$ a chi-square distribution with $j$ degrees of freedom under the assumption that $X_{1},...,X_{j}$ are statistically independent [14]. We used a nominal p-value cut-off of 1% to select the list of genes which significantly deviate from HWE. The computed HET at the gene level are then used to cluster the cattle breeds using the PermutMatrix [15] software.

**Gene Ontology (GO) enrichment analyses**

In order to test whether genome regions with above or below average levels of heterozygosity are associated with biological processes which may have relevance for breed discrimination, we performed GO enrichment analysis using GOrilla [16,17]. Gorilla is an online tool for discovering and visualizing enriched GO terms in a list of genes. In our study, the background is the list of all 8,631 genes considered. Our target lists were selected based on the genes deemed significant in each breed based on the deviation from HWE. We also considered two other gene lists which are ranked based on their PC1-based contribution to the *Bos indicus* and *Bos taurus* content.

It is expected that the significantly enriched GO terms will assist in the identification of key biological processes responsible for the human and environmentally driven evolutionary differences between *Bos taurus* and *Bos indicus* breeds. Furthermore, the genes belonging to the enriched GO terms could be crucial in explaining genetic variations in different cattle breeds.

**Functional attributes and bovine fertility related genes**

Our list of 8,631 genes was further catalogued across a series of functional attributes including transcription factors (TF), genes which are expressed in a tissue-specific (TS) manner, genes encoding secreted proteins (SE), and kinases (KI). We extracted the list of genes which are TF from the Animal Transcription Factor Database (<http://www.bioguo.org/AnimalTFDB/>) [18], genes that are TS from the Tissue-specific Gene Expression and Regulation (TIGER [19]; <http://bioinfo.wilmer.jhu.edu/tiger/>) in humans, secreted proteins from the secretome component of the Human Protein Atlas (<http://www.proteinatlas.org/humanproteome/secretome>) [20], and kinases from the Human Kinome database (<http://kinase.com/human/kinome/>) [21]. Human databases were used in the absence of similar resources for cattle; this could be improved by future annotation of the bovine genome.

Resulting from these data mining approaches, we retrieved a total of 1,160 TF, 6,612 TS, 2,919 SE, and 620 KI genes. Of these, the number of genes present in our list of 8,631 genes was 477 TF, 1,884 TS, 971 SE, and 167 KI. There were 5,740 genes not assigned to any of these four functional categories. The overlap of our list of 8,631 genes with these four categories is shown in S4 Fig(A).

Further, we retrieved a set of genes important for puberty development in beef cattle from previous reports [2-5]. Based on the research of Canovas et al. [2] and Fortes et al. [3], we collated 2,413 and 3,159 fertility genes present in their respective gene networks. Of these, we only considered those genes annotated to at least one of the four functional attributes (TF, TS, SE, KI) and included in our list of 8,631 genes. This resulted in 457 and 822 genes respectively for Canovas et al. [2] and Fortes et al. [3]. We also collated the fertility genes from two other studies, namely the 61 genes listed by Thomas et al. [4] and the 62 genes listed in the review by Fortes et al. [5]. Again, after identifying those that overlap with our list 8,631 genes, we retained 21 and 24 fertility genes from the two studies, respectively. The 1,157 unique genes corresponding to the four lists of fertility genes were distributed according to the Venn diagram of S4 Fig(B).

On further exploring the list of 1,157 fertility (FE) genes collated from the literature, we identified the four functional attributes (TF, TS, SE, KI) that are associated with them. The overlap of these functional attributes for the 1,157 fertility genes is shown in S4 Fig(C). From this set of 1,157 genes, we consider those that are TF and overlap with at least one of the other three functional attributes. This yields 61 fertility genes according to Fig. 3c. We also consider genes that have at least two functional attributes – 9 genes are SE and KI, 2 genes are TS, SE, KI, 14 genes are TS and KI. This yields a total of 86 fertility genes that we used in our analysis.

**A Co-Heterozygosity Gene Network**

We inferred a gene co-heterozygosity network based on the partial correlation and information theory (PCIT) algorithm [1] to identify significant edges. The objective was to consider genes which deviate significantly from HWE, genes that contribute strongly to the *Bos indicus* and *Bos taurus* content, genes which are related to cattle puberty, and to analyse the network structure amongst these three sets of genes. We overlaid the network with identifying information on whether the genes are TF, TS, SE, KI and/or fertility related (FE). We used Cytoscape (<http://www.cytoscape.org>) [22] to visualise and analyse the resulting network.

In particular, we place emphasis on devising the regulatory potential of the inferred network by focusing on the FE genes that appear in the network and the identity of the genes to which FE are connected. Following previously described approaches [23], a search algorithm was employed to locate the minimal trio of FE genes that span the majority of the network topology.

**References cited in S1 Methods Supporting Information**

1. Reverter A, Chan EKF (2008) Combining partial correlation and an information theory approach to the reversed engineering of gene co-expression networks. Bioinformatics 24: 2491-2497.

2. Canovas A, Reverter A, DeAtley KL, Ashley RL, Colgrave ML, et al. (2014) Multi-tissue omics analyses reveal molecular regulatory networks for puberty in composite beef cattle. PLoS One 9: e102551.

3. Fortes MR, Reverter A, Zhang Y, Collis E, Nagaraj SH, et al. (2010) Association weight matrix for the genetic dissection of puberty in beef cattle. Proc Natl Acad Sci U S A 107: 13642-13647.

4. Thomas M. SNP Discovery in RNA-Seq Across Breeds of Cattle in Puberty-Related Candidate Genes (ie, Network Hubs). Plant and Animal Genome.

5. Fortes MR, Nguyen LT, Porto Neto LR, Reverter A, Moore SS, et al. (2016) Polymorphisms and genes associated with puberty in heifers. Theriogenology 86: 333-339.

6. Bolormaa S, Pryce JE, Kemper K, Savin K, Hayes BJ, et al. (2013) Accuracy of prediction of genomic breeding values for residual feed intake and carcass and meat quality traits in Bos taurus, Bos indicus, and composite beef cattle. J Anim Sci 91: 3088-3104.

7. Mudadu MA, Porto-Neto LR, Mokry FB, Tizioto PC, Oliveira PSN, et al. (2016) Genomic structure and marker-derived gene networks for growth and meat quality traits of Brazilian Nelore beef cattle (vol 17, 235, 2016). Bmc Genomics 17.

8. Chang CC, Chow CC, Tellier LC, Vattikuti S, Purcell SM, et al. (2015) Second-generation PLINK: rising to the challenge of larger and richer datasets. Gigascience 4: 7.

9. Bertolini F, Galimberti G, Calo DG, Schiavo G, Matassino D, et al. (2015) Combined use of principal component analysis and random forests identify population-informative single nucleotide polymorphisms: application in cattle breeds. Journal of Animal Breeding and Genetics 132: 346-356.

10. Gibbs RA, Taylor JF, Van Tassell CP, Barendse W, Eversoie KA, et al. (2009) Genome-Wide Survey of SNP Variation Uncovers the Genetic Structure of Cattle Breeds. Science 324: 528-532.

11. Lewis J, Abas Z, Dadousis C, Lykidis D, Paschou P, et al. (2011) Tracing Cattle Breeds with Principal Components Analysis Ancestry Informative SNPs. Plos One 6.

12. Bolormaa S, Hayes BJ, Hawken RJ, Zhang Y, Reverter A, et al. (2011) Detection of chromosome segments of zebu and taurine origin and their effect on beef production and growth. Journal of Animal Science 89: 2050-2060.

13. McLachlan GJ, Peel D, Basford KE, Adams P (1999) The EMMIX software for the fitting of mixtures of normal and t-components. Journal of Statistical Software 4: 1-14.

14. Davis CS (1982) The Distribution of a Linear Combination of Chi-Square Variables. Biometrics 38: 279-279.

15. Caraux G, Pinloche S (2005) PermutMatrix: a graphical environment to arrange gene expression profiles in optimal linear order. Bioinformatics 21: 1280-1281.

16. Eden E, Lipson D, Yogev S, Yakhini Z (2007) Discovering motifs in ranked lists of DNA sequences. PLoS Comput Biol 3: e39.

17. Eden E, Navon R, Steinfeld I, Lipson D, Yakhini Z (2009) GOrilla: a tool for discovery and visualization of enriched GO terms in ranked gene lists. BMC Bioinformatics 10: 48.

18. Zhang HM, Chen H, Liu W, Liu H, Gong J, et al. (2012) AnimalTFDB: a comprehensive animal transcription factor database. Nucleic Acids Research 40: D144-D149.

19. Liu X, Yu XP, Zack DJ, Zhu H, Qian J (2008) TiGER: A database for tissue-specific gene expression and regulation. Bmc Bioinformatics 9.

20. Uhlen M, Fagerberg L, Hallstrom BM, Lindskog C, Oksvold P, et al. (2015) Tissue-based map of the human proteome. Science 347.

21. Manning G, Whyte DB, Martinez R, Hunter T, Sudarsanam S (2002) The protein kinase complement of the human genome. Science 298: 1912-+.

22. Shannon P, Markiel A, Ozier O, Baliga NS, Wang JT, et al. (2003) Cytoscape: a software environment for integrated models of biomolecular interaction networks. Genome Research 13: 2498-2504.

23. Reverter A, Fortes MR (2013) Breeding and Genetics Symposium: building single nucleotide polymorphism-derived gene regulatory networks: Towards functional genomewide association studies. J Anim Sci 91: 530-536.
