# Supplementary material for: The Bos taurus–Bos indicus balance in fertility and milk related genes
Source: PLoS One. 2017 Aug 1;12(8):e0181930. doi: 10.1371/journal.pone.0181930 (PMC5538644; doi:10.1371/journal.pone.0181930)
Supplement: S2 Text — (DOCX) [file pone.0181930.s002.docx]

# S2 Results and Discussion Supporting Information

# The *Bos taurus*–*Bos indicus* balance in fertility genes

Parthan Kasarapu, Laercio R. Porto-Neto, Marina R. S. Fortes, Sigrid A. Lehnert, Mauricio A. Mudadu, Luiz Coutinho, Luciana Regitano, Andrew George and Antonio Reverter

# Results and Discussion

**Principal component analysis determines the contribution of genes to the *Bos indicus* content**

The empirical distribution of SNP weights as two distinct modes requires a mathematical model, namely a mixture model with two Normal distributions, to quantify the contribution of the SNP to the *Bos indicus* content in cattle. Statistical inference of the mixture parameters leads to mean and standard deviation estimates of $(\mu_{1}=-1.26, \sigma_{1}=0.53)$ and $(\mu_{2}=0.61, \sigma_{2}=0.47)$ for the two Normal distributions respectively. The standard deviation $\sigma_{1}>\sigma_{2}$ corresponds to a flatter left mode that represents the *Bos* *indicus* component. The estimate of the mixing proportion is $w=0.31$ establishing an effective membership of 31% and 69% to the *Bos indicus* and *Bos taurus* components, respectively. We illustrated the statistical modelling of the empirical distribution of the SNP weights by overlaying with the resultant mixture model in S1 Fig. The two grey curves correspond to the individual Normal distributions and the black curve is their weighted linear combination that resulted in the mixture model. This model has enabled us to rigorously quantify the importance of a SNP to the *Bos indicus* content in the cattle genome. We aggregated the importance of individual SNP to compute the contribution to *Bos indicus* of each of the 8,631 genes surveyed in our study. This metric allowed classifying each gene that had a minimum of six SNP according to either *Bos indicu*s or *Bos taurus* ancestry.

**Gene co-heterozygosity network**

Out of the possible 823,686 connections among the 1,284 genes, PCIT identified 315,996 significant ones implying a clustering coefficient of 38.36%. S5 Fig shows the distribution of the entire connections between the 1,284 genes and those that were found to be significant. We further filtered genes based on their correlation strength as determined by PCIT at various cut-offs and were the correlation is computed for each pair-wise gene’s heterozygosity across the 19 breed populations (S1 Table). For a correlation threshold of 0.90, we obtained 858 genes with 12,958 significant connections. The degree distribution of this sub-network is shown in S6 Fig. Consistent with a scale-free network, we observed that the graph of the number of nodes and a corresponding degree using the logarithm transformation exhibits a strong linear relationship with a correlation of 0.78 (p-value < 2.2 x 10^-16^). The maximum degree is 172 and corresponds to the *CBL* proto-oncogene, a negative regulator of many signal transduction pathways which has been linked with myeloma in humans [[51-53](#_ENREF_51)]. This gene was not annotated to any of the four functional categories and it was not included in the list of fertility genes. Also, its relevance in the context of *Bos taurus – Bos indicus* axis is yet to be determined. However, its contribution to Bos indicus was estimated at 61.93% placing it in the top 10% of all 8,631 genes scrutinised in this study.

S1 Table: PCIT statistics for the 1,284 network genes across various thresholds for the co-heterozygosity correlation.

| Correlation threshold | Significant connections | Clustering coefficient (%) | Number of genes | Functional categories^A^  TF TS SE KI FE | | | | |
| --- | --- | --- | --- | --- | --- | --- | --- | --- |
| 0.00 | 315,996 | 38.36 | 1,284 | 153 | 439 | 255 | 77 | 86 |
| 0.50 | 315,287 | 38.28 | 1,284 | 153 | 439 | 255 | 77 | 86 |
| 0.55 | 311,820 | 37.86 | 1,284 | 153 | 439 | 255 | 77 | 86 |
| 0.60 | 300,487 | 36.48 | 1,282 | 153 | 438 | 254 | 76 | 86 |
| 0.65 | 273,018 | 33.15 | 1,277 | 152 | 437 | 252 | 76 | 85 |
| 0.70  0.75 | 223,657  160,379 | 27.15  19.47 | 1,268  1,250 | 151  148 | 435  429 | 251  247 | 76  75 | 85  85 |
| 0.80  0.85  0.90  0.95 | 97,378  45,882  12,958  1,098 | 11.82  5.57  1.57  0.13 | 1,227  1,130  858  328 | 145  136  108  50 | 426  392  301  116 | 242  217  166  56 | 72  68  57  23 | 84  78  66  32 |

^A^TF = transcription factor; TS = tissue specific; SE = secreted; KI = kinases; FE = fertility-related.

For the 1,284 network genes, there was no significant difference in their *Bos indicus* content when they were classified based on their functional attributes (S7 Fig). However, we observed a significantly higher Bos indicus content for the 86 FE genes as compared to the remaining 1,198 network genes. This was attributed to fertility genes being under strong selection among the various cattle lineages [[63](#_ENREF_63)]. S8 Fig(A) shows the co-heterozygosity gene network of 1,284 genes where only edges that have a significant correlation of at least 0.90 are retained. From this dense network, we retrieved sub-networks associated with fertility genes by searching for important trios of genes [[64](#_ENREF_64)]. These top trios span the overall network and can therefore aid in the identification of parsimonious networks reflecting the entire process. In essence, three FE genes with lots of connections, but many in common would not make a good far-reaching trio due to their inherent redundancy. Some examples of FE gene trios are displayed in Fig. 15. The trio formed by *GATA4, NR1H4, VAX2*, shown in S8 Fig(B), results in a sub-network that clearly distinguishes clusters of genes based on their contributions to the Bos indicus content. We observed that *GATA4* is connected to genes that have higher Bos indicus content. *GATA* itself has a high contribution to Bos indicus content of 72.2%. The gene *NR1H4* is connected to a set of genes that have moderate values of Bos indicus content, whereas the gene *VAX2* with a low Bos indicus content is connected to similar genes with extremely low Bos indicus content. Similar behaviour is observed in S8 Fig(C), where *POU2F1* with a high contribution to Bos indicus content of 56.41% is associated with a dense network of genes also with high Bos indicus content. On the other hand, the genes *ELF5* and *ROCK2* are connected to those that have a low Bos indicus content. Our analysis brings to light novel associations that could assist in the identification of genes that play an important role in biological functions of relevance to domestication events.
