# Supplementary figures and images for: The Bos taurus–Bos indicus balance in fertility and milk related genes
Source: PLoS One. 2017 Aug 1;12(8):e0181930. doi: 10.1371/journal.pone.0181930 (PMC5538644; doi:10.1371/journal.pone.0181930)

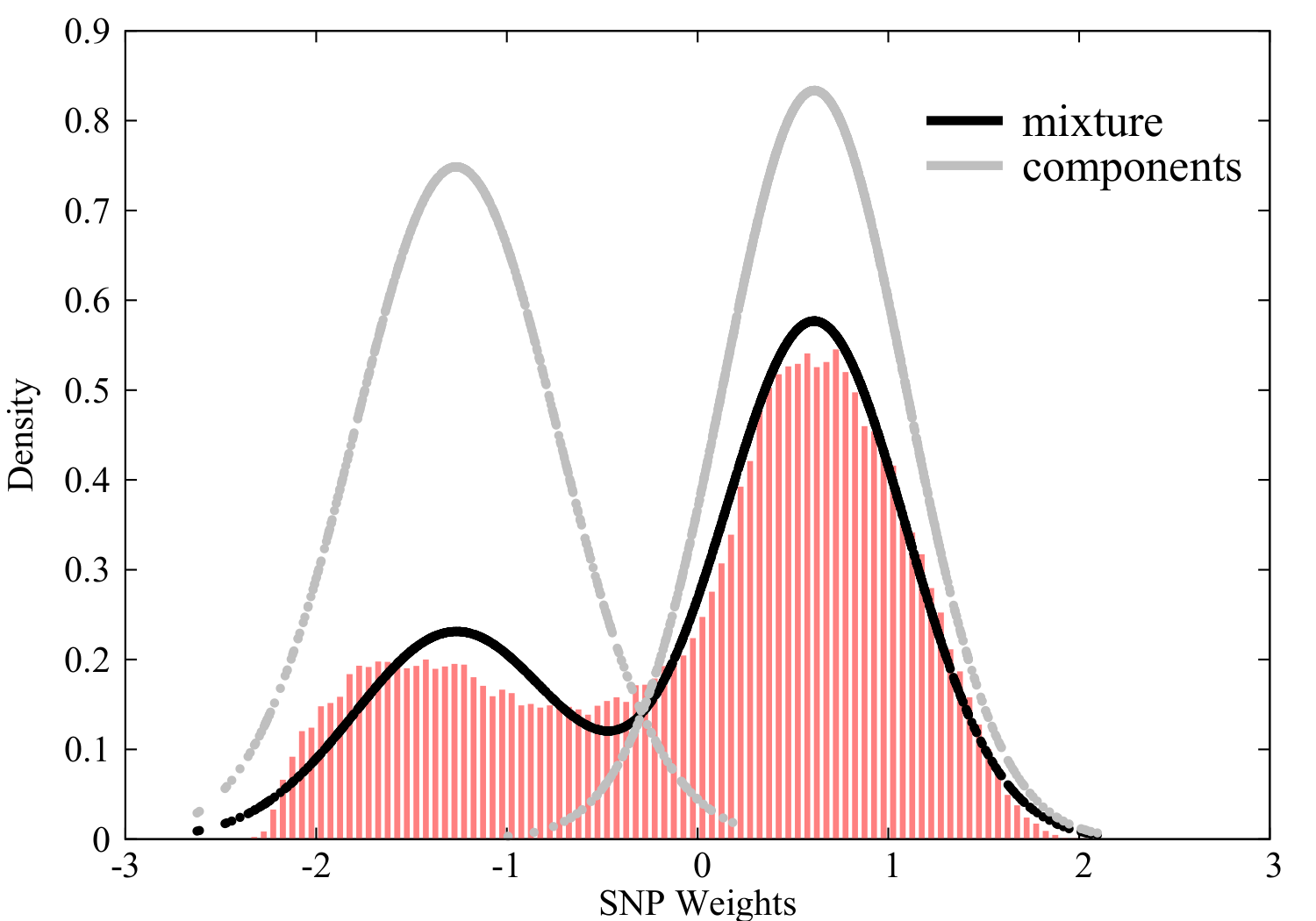

Supplement: S1 Fig — Left and Right modes describe the Bos indicus and Bos taurus components, respectively. Red indicates the actual distribution of SNP weights, grey curves are the individual Normal distributions, and black curve is the mixture model obtained by combining the two Normal distributions. (TIFF) [file pone.0181930.s004.tiff]

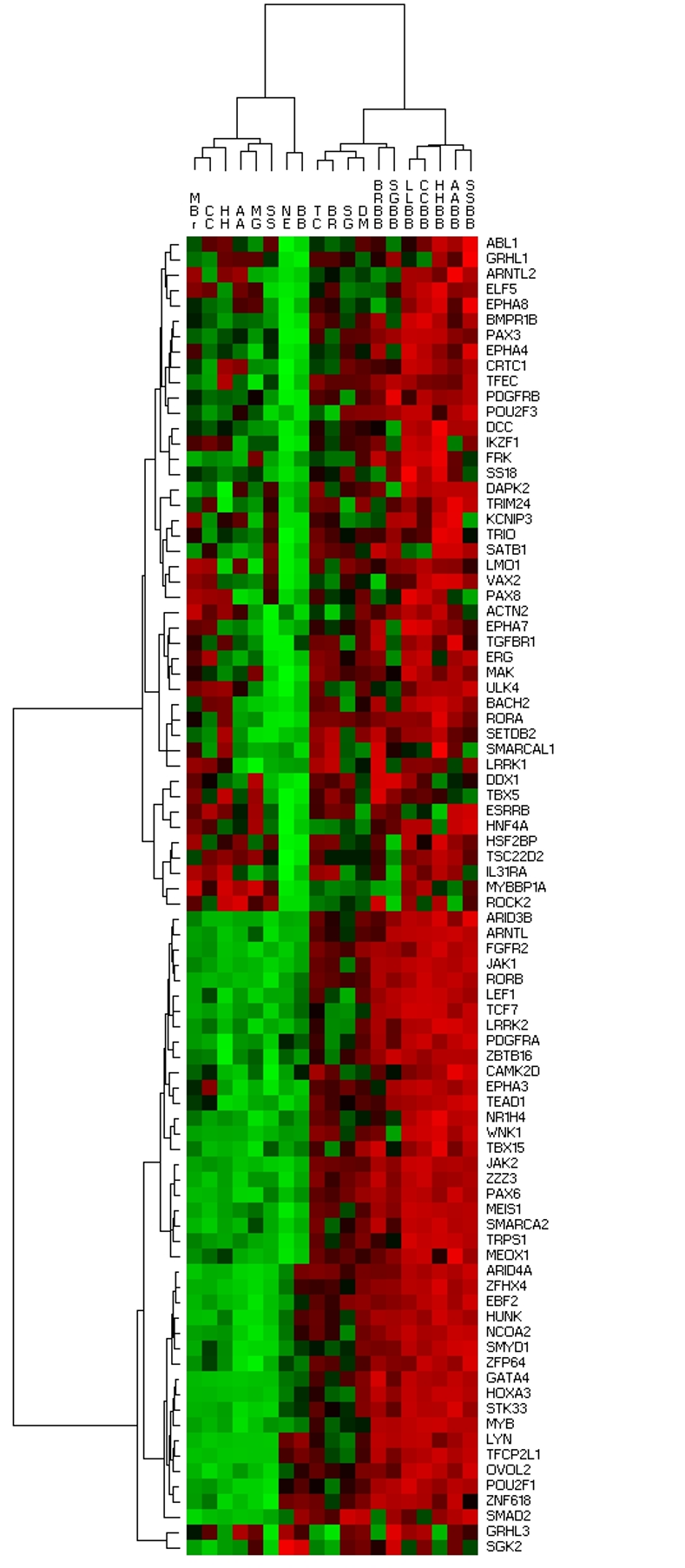

Supplement: S2 Fig — The gradient from green to black to red correspond to low, medium and high heterozygosity. (TIFF) [file pone.0181930.s005.tiff]

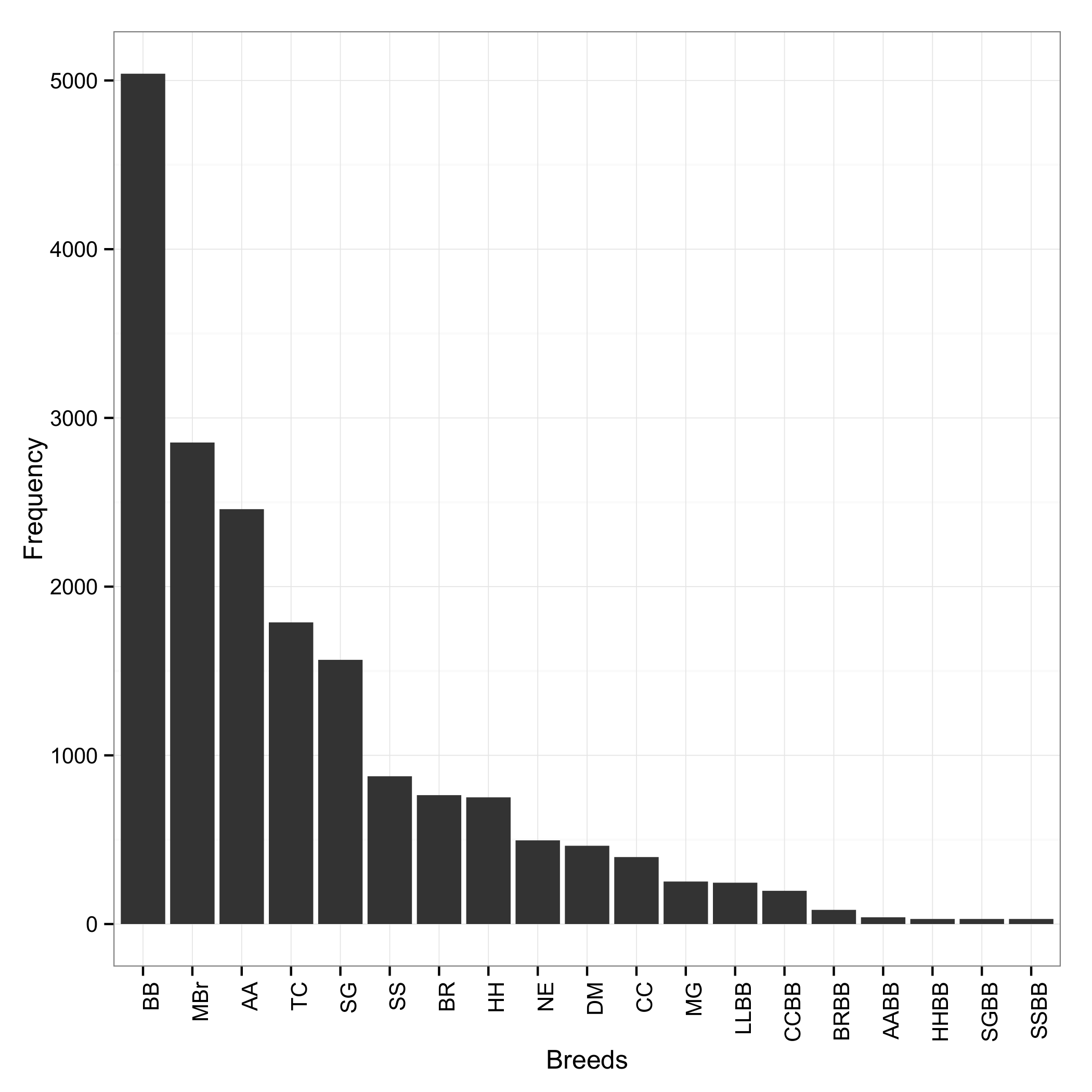

Supplement: S3 Fig — (TIFF) [file pone.0181930.s006.tiff]

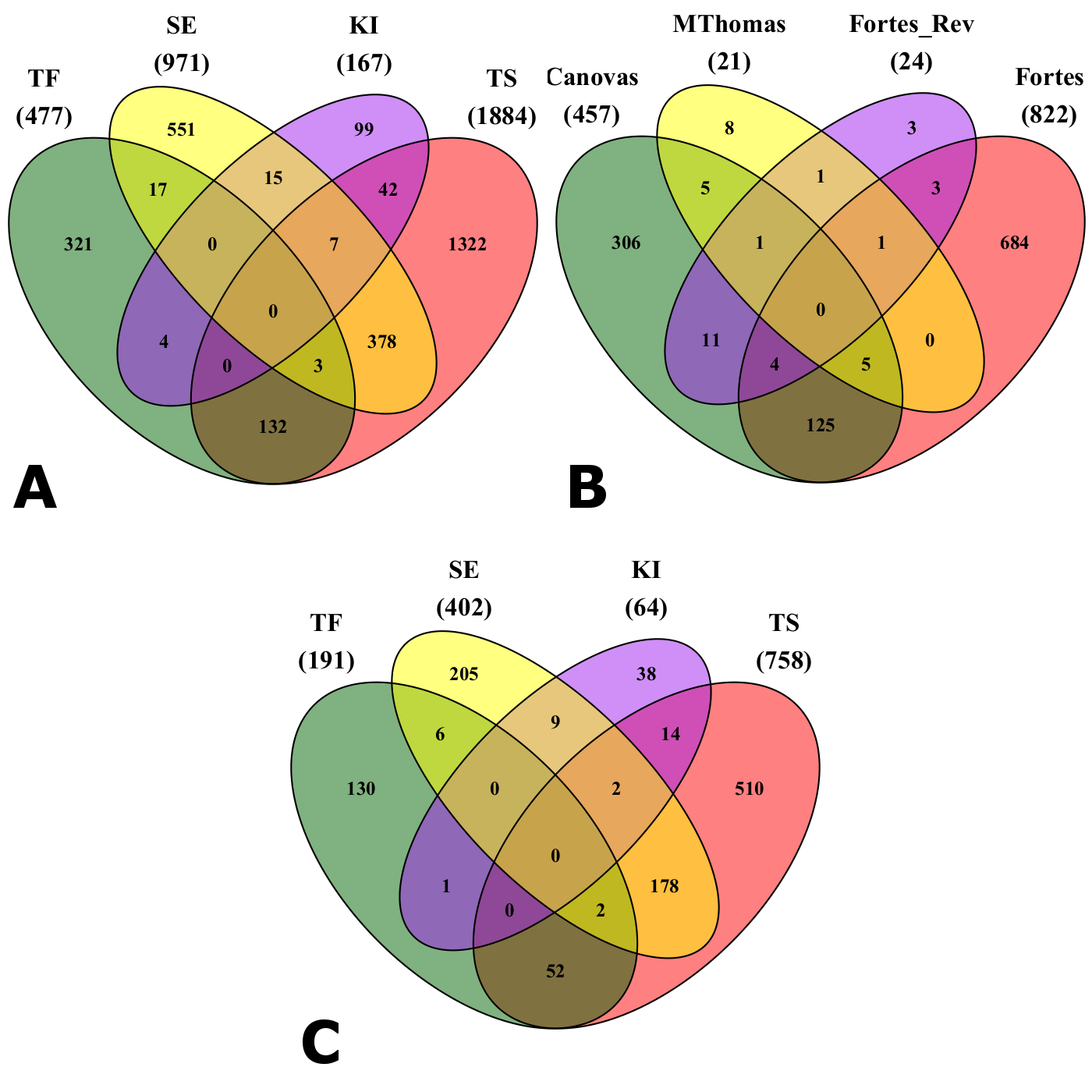

Supplement: S4 Fig — (A) The list of 2,891 genes from the entire list of 8,631 genes that belong to the four main functional categories of transcription factor (TF), secreted hormones (SE), kinases (KI) and genes expressed in tissue-specific (TS) manner; (B) The subset of 1,157 fertility genes collected from the literature where Canovas, MThomas, Fortes_Rev and Fortes correspond to [35], [37], [84] and [36]respectively; (C) The same list of 1,157 fertility genes across the four functional attributes (excluding 10 genes that are not TF, TS, SE, KI). (TIFF) [file pone.0181930.s007.tiff]

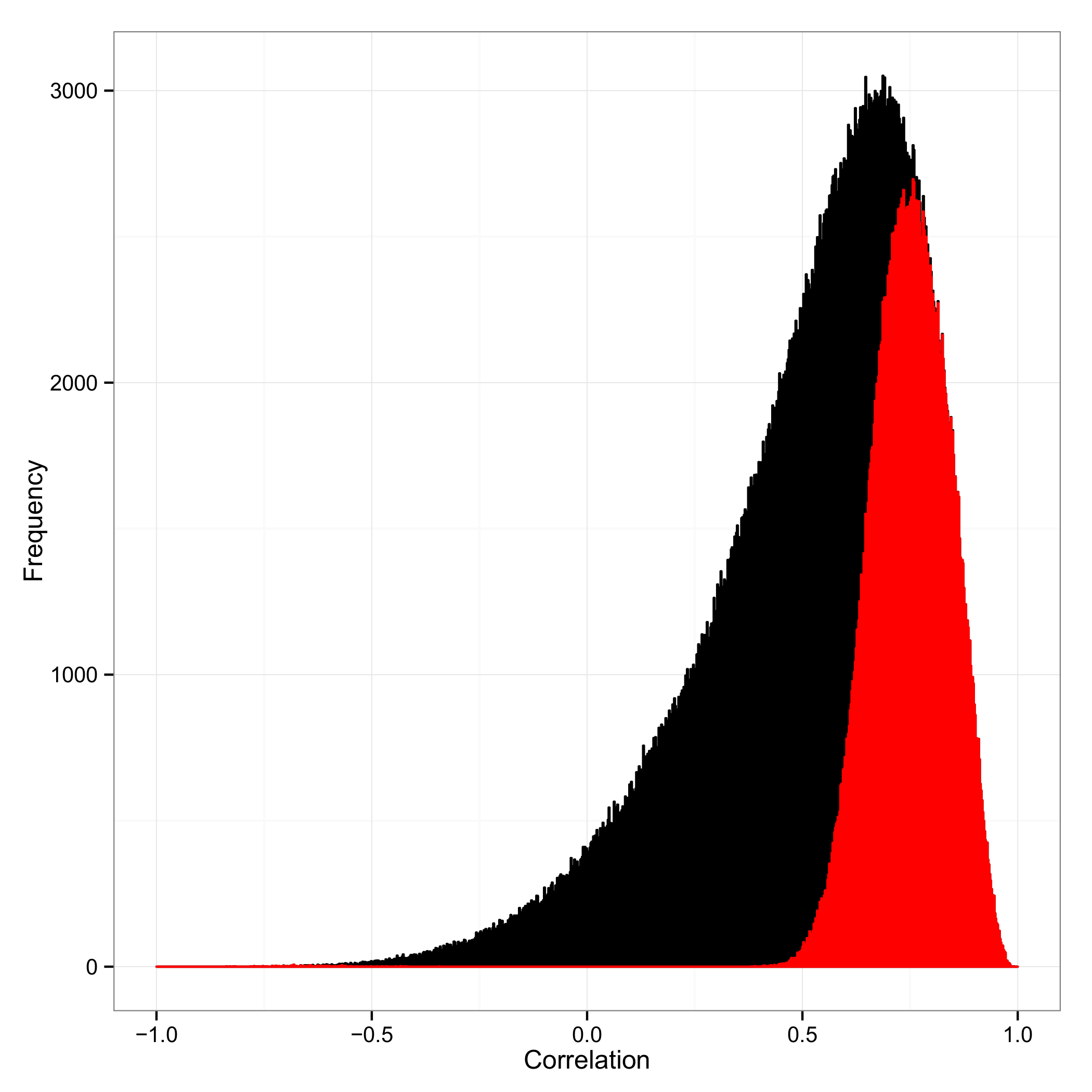

Supplement: S5 Fig — (TIFF) [file pone.0181930.s008.tiff]

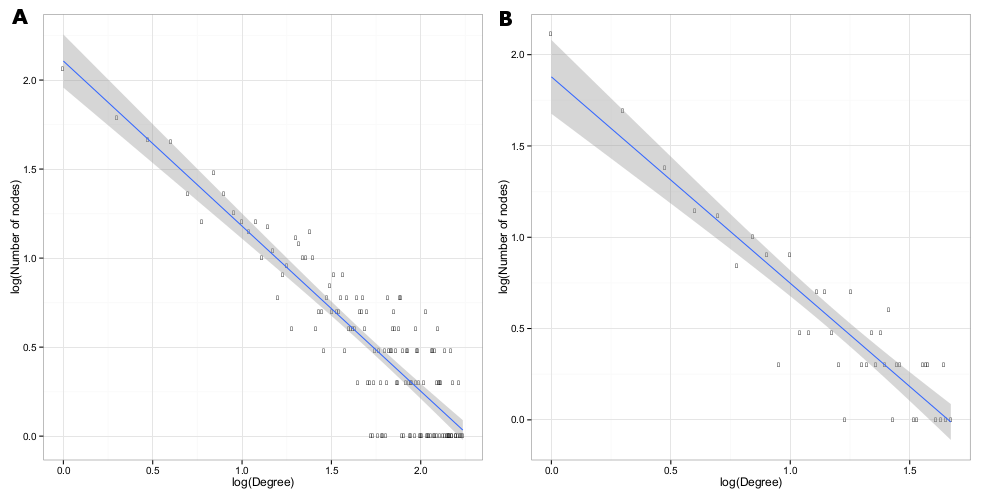

Supplement: S6 Fig — (A) At a correlation cut-off of 0.90 comprising of 858 genes and 12,958 significant connections (B) At a correlation cut-off of 0.95 comprising of 328 genes and 1,098 connections. (TIFF) [file pone.0181930.s009.tiff]

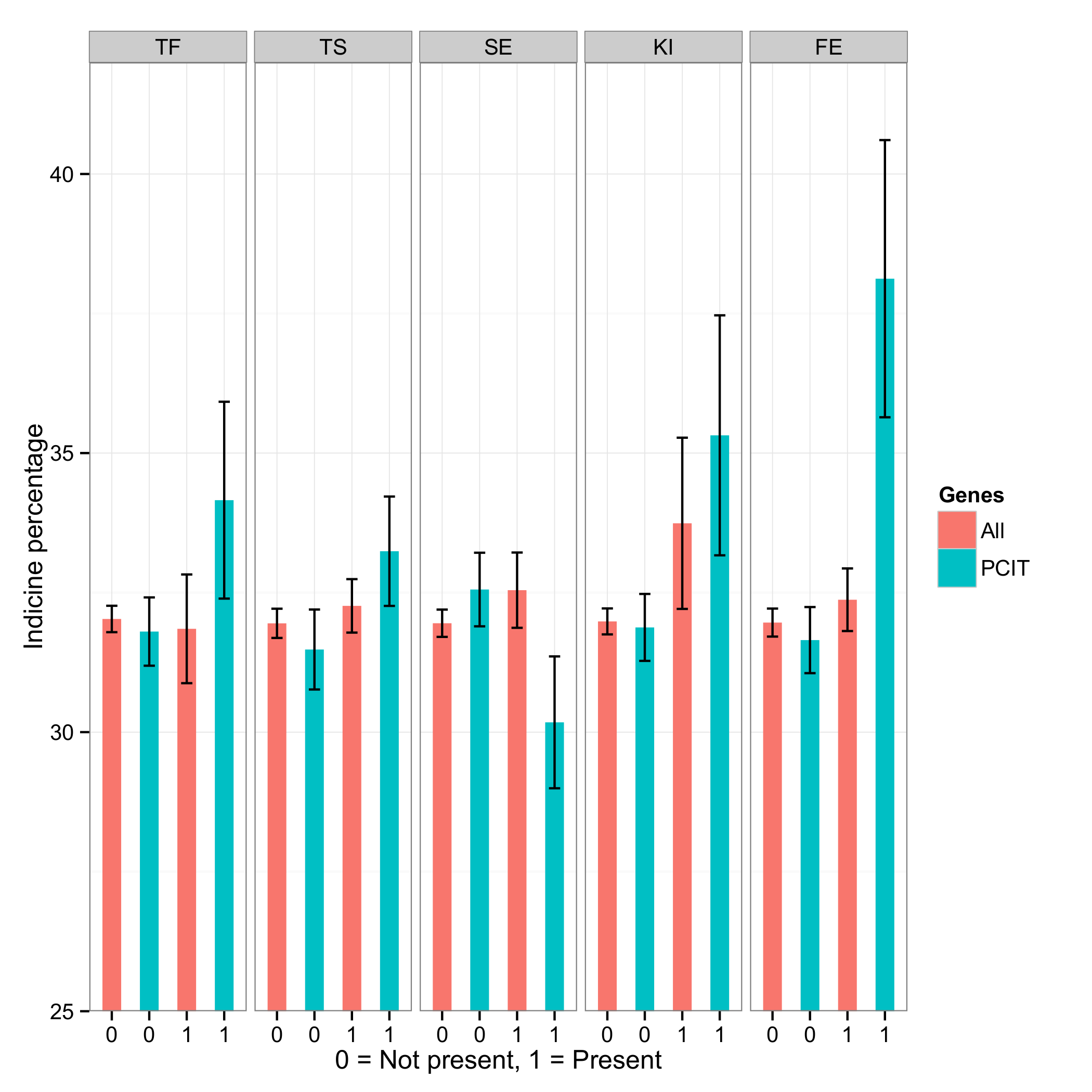

Supplement: S7 Fig — All corresponds to the 8,631 genes in our analysis and PCIT corresponds to the 1,284 network genes. The only category for which significant differences exists (p-value < 0.01) in the indicine percentage is for fertility-related genes. (TIFF) [file pone.0181930.s010.tiff]

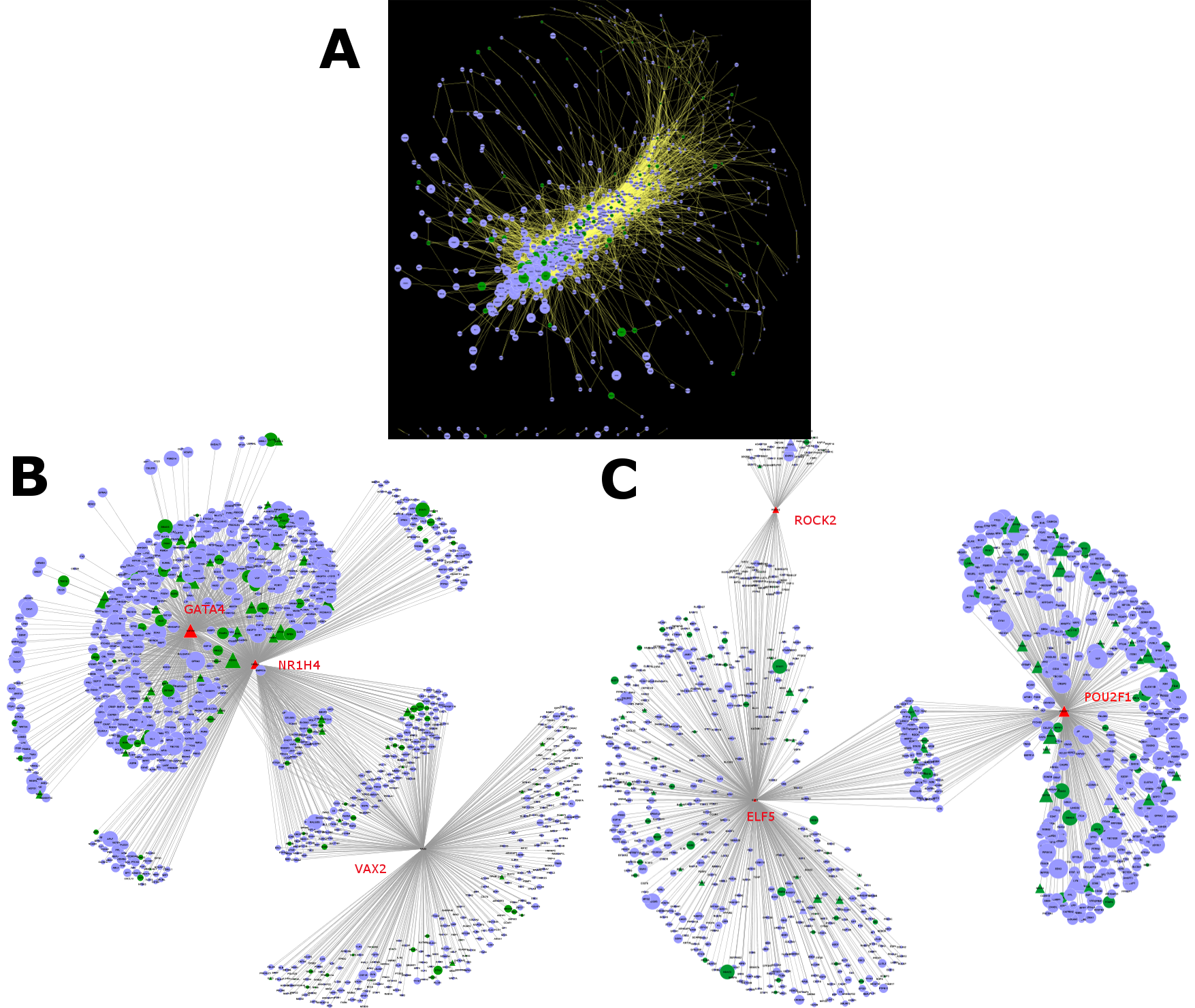

Supplement: S8 Fig — The size of the node corresponds to the indicine content. The nodes in green are transcription factors and remaining nodes in the network are purple-coloured. Nodes that are triangle-shaped are fertility-related genes and others are denoted by circles: (A) PCIT network after applying a threshold of 0.90; (B) The network spanned by the trio of fertility related genes GATA4, NR1H4, VAX2; (C) The network spanned by the trio of fertility related genes ELF5, ROCK2, POU2F1. (TIFF) [file pone.0181930.s011.tiff]
